# Spectral evidence for generic charge → acceptor interactions in carbamates and esters†

**DOI:** 10.1039/d0ra00313a

**Published:** 2020-03-24

**Authors:** Erode N. Prabhakaran, Shama Tumminakatti, Kamal Vats, Sudip Ghosh

**Affiliations:** Department of Organic Chemistry, Indian Institute of Science Bangalore Karnataka India – 560 012 eprabhak@iisc.ac.in; DOS in Organic Chemistry, University of Mysore Manasagangotri Mysore 57006 India

## Abstract

The correlations of the ^1^H NMR, ^13^C NMR and FT-IR spectral data from the R–O–C=O groups in the alkyl carbamates and esters of homologous alcohols reveal R-group-dependent negative charge stabilization at the carbonyl oxygen and their donation to generic acceptors at C^α^ of even alkyl alcohols (R), which explains several of their apparently anomalous properties.

## Abstract 2

NMR, FT-IR spectral correlations of the R–O–C=O groups in carbamates and esters of homologous alcohols (R) reveal R-group-dependent negative charge stabilization at the carbonyl oxygen and its donation to generic acceptors at Cα of even alkyl R.

## Graphical abstract

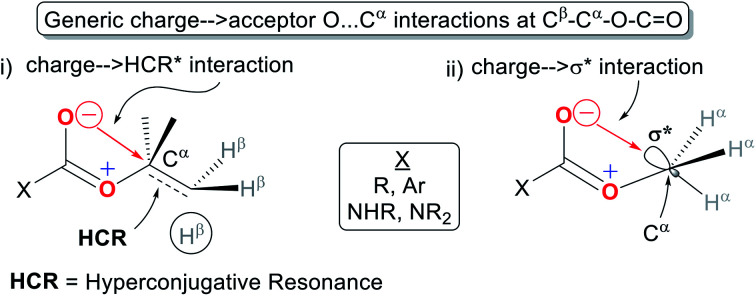

Electronic interactions at the R–O–C=O groups in carbamates and esters are less understood than that at the R–N–C=O groups in amides. Carbamates in which both these groups are fused at the carbonyl C=O bond, have several ester-like rather than amide-like features^1,2^ including comparable C–O bond lengths in crystals,^3–6^ favourable interactions between the dipoles of the C=O and O–R bonds in the predominant s-*trans* rotamers of the R–O–C=O groups and unfavourable lone pair repulsion between the two ester oxygens in their trace s-*cis* rotamers^7–10^ (Fig. 1a). However, several anomalous properties of carbamates and esters cannot be explained by these dipole and repulsive forces alone. For example, despite the electronic resonance at the O–C=O group in esters and additionally at the N–C=O group in carbamates (both of which augment the electron density at their carbonyl oxygens), their carbonyls have remarkably lower basicities than the carbonyls of amides^11^ and even those of ketones, which lack such resonance effects.^12^ The barriers to the C–N bond rotation for carbamates are 3–4 kcal mol^−1^ lower than those for analogous amides.^13,14^ Carbamate and ester carbonyl oxygens also have poor hydrogen bond accepting propensities compared to amides.^15–20^

**Fig. 1:**
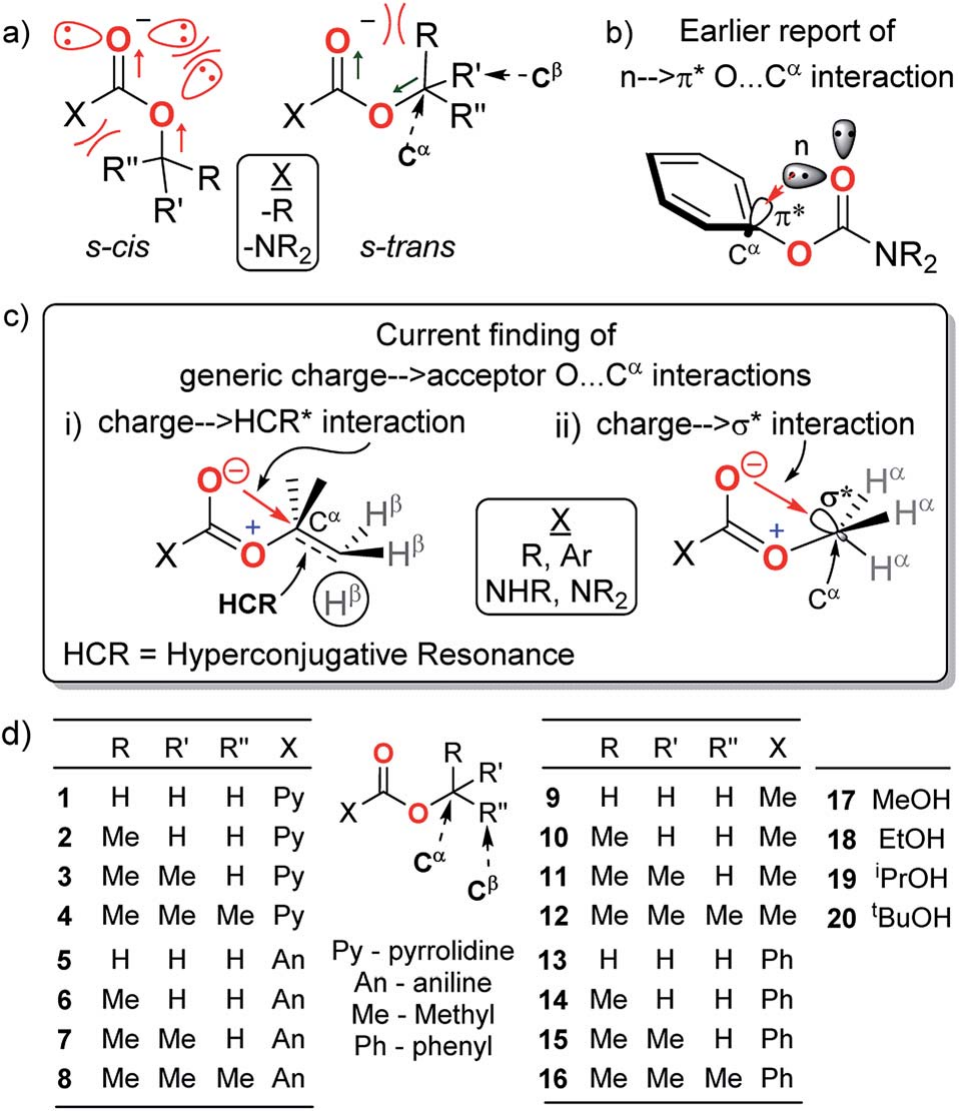
(a) Dipole–dipole, electronic and steric interactions governing the rotamer stabilities at R–O–C=O frameworks in carbamates and esters; (b) earlier report of n → π* O⋯C^α^ interaction in phenyl carbamates; (c) current finding of generic donor → acceptor interactions: (i) charge → HCR* (ii) charge → σ*; (d) list of carbamates (1–8), esters (9–16) and alcohols (17–20) investigated. HCR is hyperconjugative resonance along the C^α^–C^β^ bond of alcohol groups in carbamates and esters.

Of particular current interest is the apparent anomaly that in the crystals of phenyl carbamates, the phenyl ring plane is oriented perpendicular to the carbamate plane^21,22^ (Fig. 1b). The presence of an extraordinary n → π* orbital overlap interaction (O⋯C^α^_phenyl_) between the lone-pair electrons on the carbonyl (C=O) oxygen and the π* orbital of the phenyl ring has been proposed as the stabilizing force for this conformer based on natural bond orbital (NBO) analysis.^22^ This is interesting for several reasons: (i) despite a decrease in the stretching frequency of C=O in the FT-IR spectrum for this conformer, which indicates a decrease in the bond order and concomitant improvement in the charge density at the carbonyl oxygen, a discussion on the origin and role of such charge on this O⋯C^α^_alcohol_ interaction is lacking. Rather, the interaction is assumed to originate from the lone pair on oxygen. (ii) Our investigation of CCDC revealed that the O⋯C^α^_alcohol_ distances at the C^α^–O–C=O groups are quite non-variant (2.62–2.81 Å) for both aliphatic and aromatic carbamates and esters,^3–6,23–27^ notwithstanding the variations in the structure resolutions. These invoked the following questions: is the O⋯C^α^ interaction specific to the π* acceptors at C^α^_alcohol_? Can any antibonding orbital at C^α^_alcohol_ act as an acceptor of the electrons from carbonyl oxygen? What is the generic nature and role of this O⋯C^α^ interaction in the C^α^–O–C=O groups? Here, we present the first spectral evidence for the generic nature of the O⋯C^α^ interactions even with non-π* orbital acceptors (like σ* and hyperconjugative resonance bonds) at C^α^ in the C^α^–O–C=O groups (Fig. 1c) of a variety of model homologous aliphatic carbamates and esters (Fig. 1d). The charge at the carbonyl oxygen is interdependent on the alkoxy groups and forms charge → acceptor O⋯C^α^ interactions, which influence the rotational states of the O–C^α^ bonds in the carbamates and esters.

To explore the possibility of the O⋯C^α^ interactions at C^α^–O–C=O in aliphatic carbamates and esters, we investigated the ^1^H NMR, ^13^C NMR and FT-IR spectra of the secondary and tertiary carbamates (1–4 and 5–8), acetates (9–12) and benzoates (13–16) of homologous aliphatic alcohols (H–C^α^H_2_–OH, MeOH; CH_3_–C^α^H_2_–OH, ethanol; (CH_3_)_2_–C^α^H–OH, isopropanol; and (CH_3_)_3_–C^α^–OH, *tert*-butanol, 17–20) (Table 1) and correlated their deviations from those of their corresponding alcohols. These are ideal models to investigate the fundamental nature and origin of the O⋯C^α^ interactions because on increasing the methyl substitution at C^α^, there is a systematic increase in the C^α^ electron density, thus progressively diminishing its electron acceptor propensity. Any O⋯C^α^ donor–acceptor interactions will thus have least assistance from other local electronic effects. Evidence for the O⋯C^α^ interactions with such electronically and sterically antagonistic aliphatic C^α^ would sufficiently reveal the generality of this interaction for any non-π* acceptors. On the other hand, different carbamates and esters account for the generality of carbonyl substituent effects.

**Table 1:** Comparison of relevant ^1^H NMR, ^13^C NMR (CDCl_3_) and FT-IR spectral data (CHCl_3_) of pyrrolidine carbamates (1–4) *N*-phenyl carbamates (5–8), alkyl acetates (9–12), alkyl benzoates (13–16) and their corresponding homologous alcohols (17–20)^a^

	^13^C NMR (*δ* ppm)			*ν* (cm^−1^)	^1^H NMR (*δ* ppm)	
	O–C^α^	OC^α^–C^β^	O=C	C=O*	OC^α^–H^α^	OC^α^–C^β^–H^β^
1	52.1 (50.4)	NA (NA)	155.5	1685	3.69 (3.68)	NA (NA)
5	52.3	NA	154.1	1705	3.78	NA
9	51.6	NA	171.5	1748	3.67	NA
13	51.5	NA	165.9	1725	3.92	NA
2	60.5 (58.3)	14.6 (18.4)	155.0	1679	4.13 (3.72)	1.26 (1.24)
6	61.1	14.5	153.6	1701	4.23	1.31
10	60.5	14.2	171.4	1740	4.12	1.25
14	60.8	14.3	166.5	1720	4.33	1.33
3	67.6 (64.5)	22.1 (25.1)	154.7	1674	4.92 (4.03)	1.23 (1.20)
7	68.7	22.1	153.7	—	5.05	1.30
11	67.6	21.8	170.6	1736	4.99	1.23
15	68.3	21.9	166.1	1716	5.24	1.35
4	78.7 (69.1)	28.4 (31.2)	154.5	1683	NA (NA)	1.46 (1.27)
8	80.4	28.3	152.7	1689	NA	1.51
12	80.1	28.1	170.4	1738	NA	1.45
16	80.9	28.2	165.8	—	NA	1.58

a *FT-IR stretching frequencies; values in parentheses are for corresponding alcohols; NA means not applicable; “—” means unambiguous data could not be obtained.

The ^13^C NMR signals (Table 1) of C^α^ in homologous alcohols^28,29^ undergo a large downfield shift (by 18.7 ppm) incrementally as the number of methyl (C^β^H_3_) substituents on C^α^ increases from zero to three (Fig. 2a). There is a simultaneous downfield shift in the ^1^H NMR signal of the corresponding H^α^ by 0.55 ppm from methyl to isopropyl alcohol. Such shifts are contrary to what is expected from the increased positive induction on C^α^–H^α^ by the C^β^H_3_ substituents. They are rather due to the positive charge at C^α^ stemming from the polarization of the C^α^–O bond, which is stabilized through the hyperconjugation effect (and increasingly so) as the number of methyl substituents on C^α^ increases. Hence, the C^α^–C^β^ σ-bonds of alcohols have additional bonding from such hyperconjugative resonance (HCR).

**Fig. 2:**
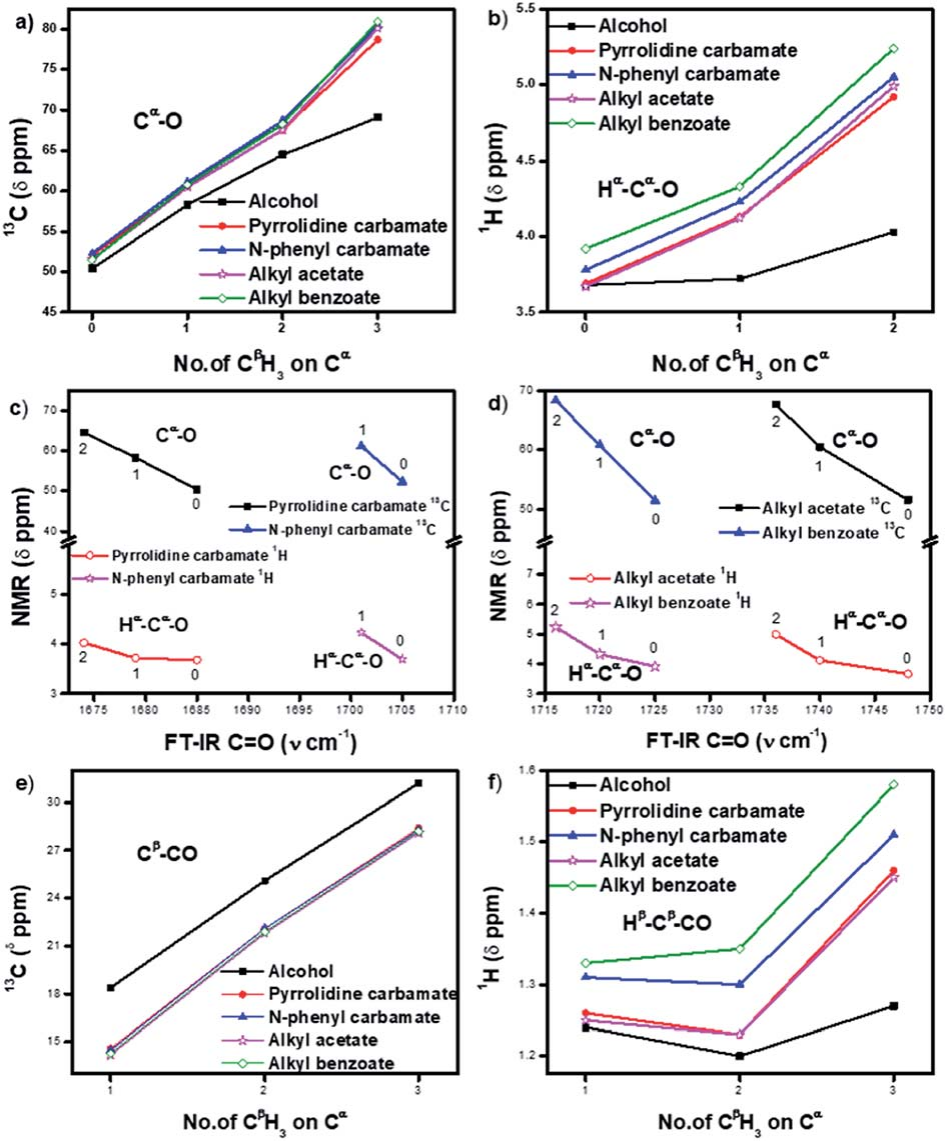
(a and b) Incremental downfield shifts in the ^13^C and ^1^H signals of H^α^ and C^α^ in carbamates (1–8) and esters (9–16) relative to the corresponding homologous alcohols (17–20), indicating the stabilization of positive charge polarization at C^α^ by hyperconjugative resonance from the C^β^H_3_ groups; (c and d) inverse correlation between ^13^C and ^1^H NMR signals of C^α^ and H^α^ and FT-IR stretching frequencies at C=O in carbamates (1–7) and esters (9–15) with increasing number of methyl (C^β^H_3_) substituents (0–2) on C^α^ (for isopropyl *N*-phenyl carbamate (7), the FT-IR value is not inserted); (e) upfield shifts in the C^β^ signals of carbamates (1–8) and esters (9–16) compared to corresponding alcohols (17–20), indicating electronic back donation towards C^β^; (f) shifts in the H^β^ signals of carbamates (1–8) and esters (9–16) compared to corresponding alcohols (17–20), indicating the dominance of charge → HCR* interactions. The lines merely indicate the trends.

In the corresponding carbamates (1–4, 5–8 (ref. 30–32)) and esters (9–12, 13–16 (ref. 33–35)), there are much larger downfield shifts for C^α^ (by 26.6 ppm for 1–4; 28.1 ppm for 5–8; 28.5 ppm for 9–12; and 29.4 ppm for 13–16) and H^α^ (by 1.23 ppm for 1–3; 1.27 ppm for 5–7; 1.32 ppm 9–11; and 1.32 ppm 13–15) compared to that for alcohols^28,29^ (Fig. 2a and b). These shifts are also incremental on increasing the C^β^H_3_ substitution on C^α^ but show steeper increase compared to that for alcohols (see ESI†). This further substantiates the hyperconjugative stabilization of greater positive polarization at C^α^ of C^α^–O–C=O, whose oxygen exists as an oxonium ion in the bipolar resonance form (C^α^–O^+^=C–O^−^) (Fig. 3). Note that if merely the electron-withdrawing induction effect of the carbonyl group was influencing the chemical shifts of C^α^ and H^α^, a constant downfield shift would have been observed on increasing the C^β^H_3_ substitution at C^α^ in either carbamates and esters compared to that for alcohols and not such incremental (steeper) downfield shifts.

**Fig. 3:**
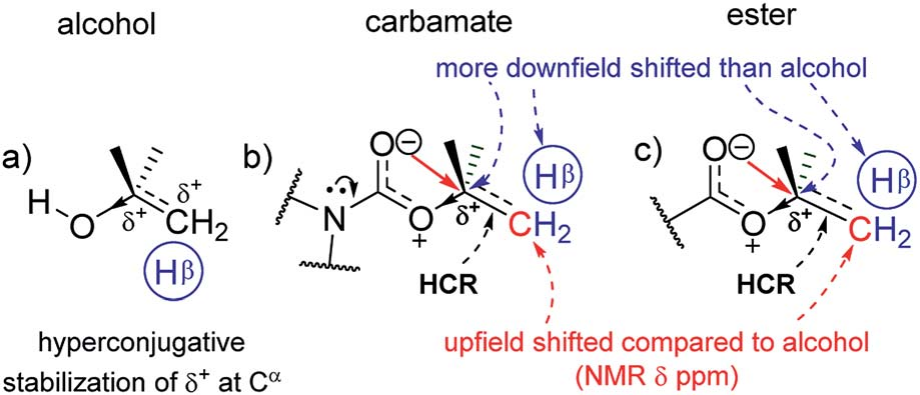
The hyperconjugation effect stabilizing the polarization at C^α^ of alkoxy groups (a) and the O⋯C^α^ electronic interactions in the s-*trans* conformer at the R–O–C=O groups in (b) carbamates and (c) esters of homologous alcohols, as observed by the correlation of their ^1^H NMR, ^13^C NMR and FT-IR data in the current work.

The remarkable uniformity in the trends of such increments in the C^α^ and H^α^ chemical shifts for the R–O–C(R′)=O groups (R′ represents primary and secondary amine, R stands for alkyl and aryl groups) of 1–16 reveals that this stabilization of the bipolar R–O^+^=C(R′)–O^−^ intermediates by the hyperconjugative effect from the R group is largely independent of the acyl substituent (R′) effects and is only slightly perturbed by the cross-conjugation from the nitrogen in R′. The longer C=O bond lengths in secondary (1.21 ± 0.01 Å)^36–40^ and tertiary (1.23 ± 0.02 Å)^5,6,24,25,41^ carbamates compared to that in acetates (1.20 ± 0.02 Å)^3,4,23,27^ and benzoates (1.21 ± 0.01 Å)^42–46^ of the homologous alcohols in the solid state (ESI†), which are reflected in their stretching frequencies (1697 ± 8 cm^−1^, 1679 ± 3 cm^−1^, 1740.5 ± 7.5 cm^−1^ and 1723 ± 7 cm^−1^, respectively) in the FT-IR spectra,^30,32,47–50^ however, clearly indicate the additional electronic resonance along the N–C=O framework. As a result, the ^13^C nuclear resonance for C=O in carbamates shifts upfield compared to that for the esters. However, since the shift is constant and independent of the number of methyl substituents on C^α^, the mixing of nitrogen lone pair with the carbonyl π-cloud does not perturb the oxonium charge state or the concomitant positive charge at C^α^. The resonance at O–C=O is hence quite strong. The shortening of the O–C single bond in the carbamates and esters, noted from the diffraction data, evidences such strong resonance at O–C=O. In fact, the lowering of the rotational energy barrier along the C–N bond in carbamates than that in the corresponding amides by 3–4 kcal mol^−1^,^13,14^ indicates the overwhelming influence of strong resonance at O–C=O in diminishing the resonance at N–C=O.

Interestingly, the FT-IR C=O stretching frequencies in the carbamates and esters consistently show an inverse correlation with the downfield shifts at their alcohol C^α^ (Fig. 2c and d), indicating interactive dependence between the negative charge at the carbonyl oxygen and the positive polarization at C^α^, an O⋯C^α^ interaction that remarkably improves on increasing the number of C^β^H_3_ substituents on C^α^.

Such O⋯C^α^ interactions are clearly evident in the crystal structures of the carbamates^5,6,24,25,36–41^ and esters^3,4,23,27,42–46^ (1–16) (ESI†), especially when there are methyl (C^β^H_3_) substituents on C^α^: (i) the C^α^–C^β^ bond is consistently oriented antiperiplanar to C=O with an angle of incidence of 160 ± 5 deg for oxygen. Note that in addition to the σ-bond along C^α^–C^β^, the current results have established the presence of HCR bonding. Hence, the O⋯C^α^ charge donation can be either to σ* or HCR* along the C^α^–C^β^ bond. (ii) Only in methyl carbamates and esters (which lack C^β^), such antiperiplanar orientation of oxygen (159 ± 5 deg) is observed with a C^α^–H^α^ σ-bond. (iii) The O⋯C^α^ distances are comparable (2.73 ± 0.02 Å) (within errors of estimation). The distances however increase slightly from 2.62 ± 0.01 Å to 2.81 ± 0.00 Å for methyl to *t*-butyl carbamates and esters due to increased O⋯C^α^ steric repulsions. (iv) All the atoms of the O=C–O–C^α^–C^β^ group are in plane, indicating lack of distortions due to the steric clashes between the C=O and R groups. There is slight deviation of the C–O–C^α^–C^β^ torsion from planarity (by ∼25 deg) only in the isopropyl carbamates and esters where the carbonyl oxygen is asymmetrically staggered between the small H^α^ and the bulky C^β^H_3_ of the isopropyl group. This slight deviation is reflected in the consistent minor deviations of their NMR and FT-IR data from the trends of their corresponding homologues. (v) The bond angles at the R–O–C=O framework are comparable in the carbamates and esters for all the homologous R groups.

The antiperiplanar orientation of C=O to the C^α^–C^β^ bond in the carbamates and esters in the solid state and the O⋯C^α^ interactions suggested by spectral indicators were consistent with each other. Interestingly, the C^β^ signals in the ^13^C NMR spectra of the carbamates and esters identically shifted upfield on increasing the C^β^H_3_ substitution at C^α^ (Fig. 2e) compared to that for alcohols; this was in contrast to the C^α^ signals, which shifted down-field. However, the H^β^ signals in the ^1^H NMR spectra showed downfield shifts of decreasing steepness with decrease in the electronic charge at the carbonyl oxygen in the order alkyl benzoates > *N*-phenyl carbamate > acetates/pyrrolidine carbamates (Fig. 2f). These indicate the predominance of the charge → HCR* (O⋯C^α^–C^β^) interaction (Fig. 1c(i)) over the charge → σ* (along C^α^–H^α^ or C^α^–C^β^) interaction and slightly greater electronic back donation of the charge from C=O to C^α^–C^β^ in the acetates and pyrrolidine carbamates (Fig. 1c(ii)). There were consistent slight deviations in particularly the *δ* ppm values of H^β^ for all the isopropyl carbamate and ester analogues (3, 7, 11, 15), as observed from the trends obtained for the remaining homologues. This was consistent with the small distortions away from the ideal anti-periplanarity of the carbonyl oxygen and the C^α^–C^β^ bond observed in their crystal structures, which diminished the charge → HCR* donation and would not be observed unless charge → HCR* predominated over charge → σ* in O⋯C^α^.

The generic charge → acceptor O⋯C^α^ electronic back donation interactions explain the X-ray structures of phenyl carbamates,^21^ where the inclusive plane of the carbamate group is perpendicular to the plane of the phenyl ring.^22^ Current data additionally indicate that this interaction is (a) largely charge → π* in nature rather than n → π*; (b) primarily localized between O and C^α^ of the phenyl ring; and (c) a consequence of the general O⋯C^α^ charge → acceptor electronic interactions at the R–O–C=O groups, which are observed for a variety of C^α^ acceptors: (1) σ* acceptor in C^α^–H^α^, when R is a methyl group (Fig. 1c(ii)); (2) HCR* acceptor in C^α^–C^β^, when R has a C^β^H group (Fig. 1c(i)); and (3) π* acceptor, when R is phenyl (Fig. 1b). In other words, the O⋯C^α^ electronic charge → acceptor back donation interaction is observed at alkyl carbamates and esters as well as they are observed in phenyl carbamate.

Note that only a charge (rather than a lone pair) donor at the carbonyl oxygen, which has a longer coulombic interaction range, is consistent with the reported observations for phenyl carbamates^22^ that the rest of the π*-orbitals of the phenyl ring (other than at its C^α^) that are at distances longer than is conducive for any n → π* orbital overlap interactions also accept electron density from the carbonyl oxygen. Moreover, the constancy (rather than decrease) in the O⋯C^α^ interactions despite increasing the substitution at C^α^ and the concomitant upfield shift in the C^β^ signals of the current analogues and the O⋯C^α^–C^β^ periplanarities with little perturbation in the O⋯C^α^ distances in crystals all substantiate an R group-dependent stabilization of the negative charge on the carbonyl oxygen, which interacts back with C^α^ of R and influences the rotational states of the O–C^α^ bond at the R–O–C=O groups. Thus, the charge → acceptor O⋯C^α^ interaction is generic to the C^α^–O–C=O group and influences the biasing of the rotational states along the O–C^α^ bond. The incorporation of the corresponding force fields in the computational methods will benefit energy minimization of the rotational states in these molecules. The generic charge → HCR*/σ*/π* interaction model at C^α^–O–C=O is also consistent with masking the charge at the carbonyl oxygen, hence explaining the low basicities and poor hydrogen bond acceptor propensities of the carbamates and esters.

Finally, it is possible that the O⋯C^α^ interaction has an electrostatic component as well due to the polarized charge at C^α^. Although this may influence the planarity of R–O–C=O even when R is a methyl group, the resonance at O–C=O would have a major role in such planarity. Moreover, such electrostatic interactions are insufficient by themselves to explain the observed C=O⋯C^α^–C^β^ anti-periplanarities and the relative downfield shifting of H^β^ (compared to alcohols). Note that the latter data also discount the possibility of O⋯H^α^–C^α^ or O⋯H^β^–C^β^-type hydrogen bonding interactions.

## Conclusions

Correlations between the ^1^H NMR, ^13^C NMR and FT-IR spectral data for the C^α^–O–C=O groups of the model carbamates and esters of homologous alcohols reveal the presence of charge → HCR*/σ* donor/acceptor O⋯C^α^ interactions along with other structural and electronic features, which explain several of the apparently anomalous properties of carbamates and esters. First, there is positive charge polarization at the C^α^ carbon attached to the alcohol oxygen, which is stabilized incrementally by hyperconjugative resonance (HCR) with increasing number of C^β^H_3_ substituents on it. Interestingly, the resulting dipolar resonance at O–C=O is comparable for carbamates and esters and is slightly perturbed by the cross-resonance from the (secondary and tertiary) carbamate nitrogen. Rather, the latter is weakened, explaining the lowering of the transition energy barrier for *cis*/*trans* isomerism at the carbamate C–N bonds compared to that for amides. The electronic charge at the carbonyl oxygen of carbamates and esters interacts back with the hyperconjugative resonance (HCR*) along C^α^–C^β^ or with σ* along C^α^–H^α^, as substantiated by the NMR and FT-IR spectral shifts (compared to those for the corresponding alcohols). Such an interaction regulates the rotational states along the O–C^α^ bonds, as seen from the C=O⋯C^α^–C^β^ and C=O⋯C^α^–H^α^ anti-periplanarities in the crystal structures of carbamates and esters. Deviations in the anti-periplanarities are directly reflected through spectral shifts. This charge → acceptor O⋯C^α^ interaction thus occurs with any (HCR*, σ* or π*) acceptor at C^α^ and explains several unique features of the C^α^–O–C=O groups: (1) the unusual orthogonality observed between the planes of phenyl and carbamate groups in phenyl carbamates and (2) the diminished basicities and hydrogen bond accepting propensities of the carbamate and ester carbonyl oxygens compared to that for ketones and amides. This is the first insight into the strong interdependence between the alcohol group and the charge at the carbonyl group of the C^α^–O–C=O groups of carbamates and esters, resulting in charge-stabilizing generic charge → acceptor O⋯C^α^ interactions that bias the rotational states of the O–C^α^ bond. Apart from providing a better understanding of the interactions in the R–O–C=O groups, the current results will help in chemical biology research^51^ and drug design,^52–54^ where carbamates play an important role.

## Conflicts of interest

There are no conflicts to declare.

## Supplementary Material

RA-010-D0RA00313A-s001

## References

[cit1] Oki M., Nakanishi H. (1971). Bull. Chem. Soc. Jpn..

[cit2] Oki M., Nakanishi H. (1970). Bull. Chem. Soc. Jpn..

[cit3] Barrow M. J., Cradock S., Ebsworth E., Rankin D. W. (1981). J. Chem. Soc., Dalton Trans..

[cit4] Boese A. D., Kirchner M., Echeverria G. A., Boese R. (2013). ChemPhysChem.

[cit5] Sepehrnia B., Ruble J., Jeffrey G. (1987). Acta Crystallogr., Sect. C: Cryst. Struct. Commun..

[cit6] Bracher B. H., Small R. (1967). Acta Crystallogr., Sect. C: Cryst. Struct. Commun..

[cit7] Le Fevre R., Sundaram A. (1962). J. Chem. Soc..

[cit8] O'Gorman J. M., Shand Jr W., Schomaker V. (1950). J. Am. Chem. Soc..

[cit9] Wilmshurst J. (1957). J. Mol. Spectrosc..

[cit10] Kozak N., Nizel'skii Y. N. (1987). J. Struct. Chem..

[cit11] Rzepa H. (2015). The Winnower.

[cit12] Filgueiras C. A., Huheey J. E. (1976). J. Org. Chem..

[cit13] Cox C., Lectka T. (1998). J. Org. Chem..

[cit14] Deetz M. J., Forbes C. C., Jonas M., Malerich J. P., Smith B. D., Wiest O. (2002). J. Org. Chem..

[cit15] Böhm H. J., Brode S., Hesse U., Klebe G. (1996). Chem.–Eur. J..

[cit16] Nobeli I., Price S., Lommerse J., Taylor R. (1997). J. Comput. Chem..

[cit17] Le Questel J.-Y., Laurence C., Lachkar A., Helbert M., Berthelot M. (1992). J. Chem. Soc., Perkin Trans. 2.

[cit18] Besseau F., Laurence C., Berthelot M. (1994). J. Chem. Soc., Perkin Trans. 2.

[cit19] Besseau F., Luçon M., Laurence C., Berthelot M. (1998). J. Chem. Soc., Perkin Trans. 2.

[cit20] Ziao N., Laurence C., Le Questel J.-Y. (2002). CrystEngComm.

[cit21] Modarresi-Alam A. R., Nowroozi A., Najafi P., Movahedifar F., Hajiabadi H. (2014). J. Mol. Struct..

[cit22] Singh S. K., Mishra K. K., Sharma N., Das A. (2016). Angew. Chem., Int. Ed..

[cit23] Baillargeon P., Dory Y. L. (2009). Cryst. Growth Des..

[cit24] Staples R. J., Gingold J. A. (2009). Z. Kristallogr.–New Cryst. Struct..

[cit25] Calleri M., Chiari G., Villa A. C., Manfredotti A. G., Guastini C., Viterbo D. (1977). Acta Crystallogr., Sect. B: Struct. Crystallogr. Cryst. Chem..

[cit26] Bursavich M., Fronczek F. (1997). Acta Crystallogr., Sect. C: Cryst. Struct. Commun..

[cit27] Mouhib H., Jelisavac D., Stahl W., Wang R., Kalf I., Englert U. (2011). ChemPhysChem.

[cit28] Fulmer G. R., Miller A. J., Sherden N. H., Gottlieb H. E., Nudelman A., Stoltz B. M., Bercaw J. E., Goldberg K. I. (2010). Organometallics.

[cit29] Babij N. R., McCusker E. O., Whiteker G. T., Canturk B., Choy N., Creemer L. C., Amicis C. V. D., Hewlett N. M., Johnson P. L., Knobelsdorf J. A. (2016). Org. Process Res. Dev..

[cit30] Borah A. J., Phukan P. (2012). Tetrahedron Lett..

[cit31] Ghosh R., Nethaji M., Samuelson A. G. (2005). J. Organomet. Chem..

[cit32] Chankeshwara S. V., Chakraborti A. K. (2006). Tetrahedron Lett..

[cit33] Abraham R. J., Bardsley B., Mobli M., Smith R. J. (2005). Magn. Reson. Chem..

[cit34] Pramanik S., Reddy R. R., Ghorai P. (2015). Org. Lett..

[cit35] Hosseini-Sarvari M., Sodagar E. (2013). C. R. Chim..

[cit36] Laidlaw R., Miura Y., Panetta C., Metzger R. (1988). Acta Crystallogr., Sect. C: Cryst. Struct. Commun..

[cit37] Goodman M., Ganis P., Avitabile G., Migdal S. (1971). J. Am. Chem. Soc..

[cit38] Nethaji M., Pattabhi V. (1985). Proceedings of the Indian Academy of Sciences.

[cit39] Singh M. K., Gangwar M., Kumar D., Tilak R., Nath G., Agarwal A. (2014). Med. Chem. Res..

[cit40] Zhang X., Liu Z., Gao Y., Li F., Tian Y., Li C., Jia X., Li J. (2018). Adv. Synth. Catal..

[cit41] Bursavich M., Fronczek F. (1997). Acta Crystallogr., Sect. C: Cryst. Struct. Commun..

[cit42] Yakovenko A. A., Gallegos J. H., Antipin M. Y., Masunov A., Timofeeva T. V. (2011). Cryst. Growth Des..

[cit43] Diskin-Posner Y., Dahal S., Goldberg I. (2000). Chem. Commun..

[cit44] Diskin-Posner Y., Patra G. K., Goldberg I. (2001). Eur. J. Inorg. Chem..

[cit45] Endo K., Ezuhara T., Koyanagi M., Masuda H., Aoyama Y. (1997). J. Am. Chem. Soc..

[cit46] Zou P., Xie M. H., Wu H., Liu Y. L., Chen Z. P. (2011). Acta Crystallogr., Sect. E: Struct. Rep. Online.

[cit47] Nolin B., Jones R. N. (1956). Can. J. Chem..

[cit48] Sundaraganesan N., Joshua B. D. (2007). Spectrochim. Acta, Part A.

[cit49] Soderquist J. A., Rosado I., Marrero Y., Burgos C. (2001). ARKIVOC.

[cit50] Jia X.-S., Wang H.-L., Huang Q., Kong L.-L., Zhang W.-H. (2006). J. Chem. Res..

[cit51] Shi Z., Griffin J. H. (1993). J. Am. Chem. Soc..

[cit52] Ferraz de Souza W., Kambe N., Sonoda N. (1996). J. Phys. Org. Chem..

[cit53] Takayama H., Shirakawa S., Kitajima M., Aimi N., Yamaguchi K., Hanasaki Y., Ide T., Katsuura K., Fujiwara M., Ijichi K. (1996). Bioorg. Med. Chem. Lett..

[cit54] Ghosh A. K., Brindisi M. (2015). J. Med. Chem..

