# Supplementary material for: Spectral evidence for generic charge → acceptor interactions in carbamates and esters
Source: RSC Adv. 2020 Mar 24;10(20):11871–5. doi: 10.1039/d0ra00313a (PMC9050606; doi:10.1039/d0ra00313a)
Supplement: RA-010-D0RA00313A-s001 [file RA-010-D0RA00313A-s001.pdf]

Electronic Supplementary Material (ESI) for RSC Advances.
This journal is © The Royal Society of Chemistry 2020

**Manuscript ID:**

**Manuscript title:**

# Spectral evidence for electronic interactions in the s-trans form of esters and carbamates

**Authors**: Erode N. Prabhakaran[a]*, Shama Tumminakatti[b], Kamal Vats[a], Sudip Ghosh[a]

**Affiliation**: [a] Department of Organic Chemistry, Indian Institute of Science
Bangalore, Karnataka 560012, India
[b] Department of Studies in Organic Chemistry, University of Mysore,
Manasagangothri, Mysuru, Karnataka 570006, India

**E-mail**: eprabhak@orgchem.iisc.ernet.in; erodeprabhakaran02@gmail.com.
Tel.: +91 80 2293 3380; Fax: (+) 91 80 2360 0529

# Table of Contents


| Item | Starting Page |
|---|---|
| **1. Manuscript title, authors list, affiliation, E-mail.** | **S1** |
| **2. Table of Contents.** | **S2** |
| **3. Supporting Information.** | |
| **-S1-**Experimental Section. | **S3** |
| **-S1.1.**Materials and Methods | **S3** |
| **4. -S2. Spectral Details** | **S4** |
| **-S2.1.** Methyl pyrrolidine-1-carboxylate **(1)** | |
| **-S2.2.** Ethyl pyrrolidine-1-carboxylate **(2)** | |
| **-S2.3.** Isopropyl pyrrolidine-1-carboxylate **(3)** | |
| **-S2.4.** *tert*-Butyl pyrrolidine-1-carboxylate **(4)** | |
| **5. -S3. $^1$H NMR and $^{13}$C NMR Spectrum** | **S7** |
| **-S3.1. Figure 1.** $^1$H NMR of **1** in $CDCl_3$ (400 MHz, 10 mmol) | |
| **-S3.2. Figure 2.** $^{13}$C NMR of **1** in $CDCl_3$ (100 MHz, 60 mmol) | |
| **-S3.3. Figure 3.** $^1$H NMR of **2** in $CDCl_3$ (400 MHz, 10 mmol) | |
| **-S3.4. Figure 4.** $^{13}$C NMR of **2** in $CDCl_3$ (100 MHz, 60 mmol) | |
| **-S3.5. Figure 5.** $^1$H NMR of **3** in $CDCl_3$ (400 MHz, 10 mmol) | |
| **-S3.6. Figure 6.** $^{13}$C NMR of **3** in $CDCl_3$ (100 MHz, 60 mmol) | |
| **-S3.7. Figure 7.** $^1$H NMR of **4** in $CDCl_3$ (400 MHz, 10 mmol) | |
| **-S3.8. Figure 8.** $^{13}$C NMR of **4** in $CDCl_3$ (100 MHz, 60 mmol) | |
| **6. -S4. Figure 9. Stacked IR (C=O stretching frequency region) of 1 to 4 ($CHCl_3$, 10 mmol)** | **S11** |
| **7. -S7. Table 6. Structural parameters at the R-O-C=O register (s-trans conformation) in carbamates and esters from corresponding crystal structures.** | **S23** |


.

## Supporting Information:

## -S1- Experimental Section:

### -S1.1. Materials and Methods.

All the reactions were performed in oven dried apparatus and were stirred using magnetic stir bars. Column chromatography was performed on silica gel (100-200μm). TLC was carried out on Kieselgel coated on aluminium sheets. Compounds were visualized by one of the (or all of the) following methods: (1) fluorescence quenching, (2) spray with a 0.2% (w/v) ninhydrin solution in absolute ethanol, (3) spray with 1% $H_2SO_4$ solution in EtOH/$H_2O$ (1:5 v/v), (4) charring on hot plate. Ethylacetate and hexanes (or petroleum ether) were obtained from Sd-fine chemicals and were fractionally distilled at their respective boiling points, before use. Dichloromethane was dried by distillation over $P_2O_5$. NMM was distilled over $CaH_2$. NMR spectra were recorded on 400 MHz spectrometers in $CDCl_3$. Chemical shifts are expressed in parts per million (ppm) from the residual non-deuterated chloroform in $CDCl_3$ ($\delta_H$ = 7.26, $\delta_C$ = 77.00). *J* values are in Hz. Multiplicities are indicated using the following abbreviations: s (singlet), d (doublet), dd (doublet of doublets), t (triplet), q (quartet), quin (quintet), hept (heptet), m (multiplet), bs (broad singlet). Infrared (IR) spectra were recorded in a FT/IR spectrometer, for thin-films (0.1 mmol) made from solutions in $CHCl_3$ (10 mmol) on sodium chloride plates or in neat (KBr pellets), with frequencies given in reciprocal centimetres ($cm^{-1}$). High resolution mass spectra were obtained by the ESI technique.

## -S2. Spectral Details:

### -S2.1. Methyl pyrrolidine-1-carboxylate (1):

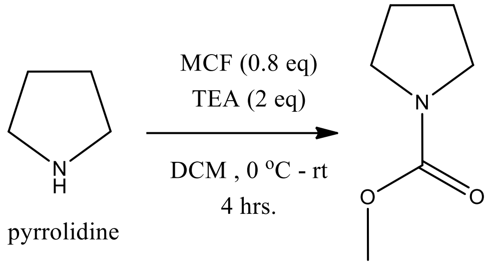


**(1)**

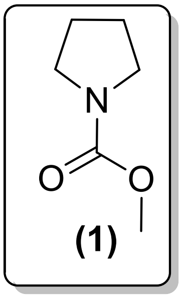

To a cold (0 °C) solution of **pyrrolidine** (500 mg, 7.03 mmol) in 14 mL Dichloromethane (DCM) added Methyl chloroformate (MCF) (435 µL, 5.62 mmol), triethylamine ($Et_3N$) (1.95 mL, 14.1 mmol) and the mixture was allowed to stir at 0 °C for 10 min, then allowed to stir at rt for 4 h. Removal of solvent resulted in a residue which was dissolved in ethyl acetate (EtOAc) (10 mL), washed with 10 mL water (2 X 5 mL) and 1N HCl solution (2 X 5 mL) and saturated $NaHCO_3$ solution (2 X 5 mL) and dried over anhydrous sodium sulphate ($Na_2SO_4$), and the organic layer was concentrated to get a residue which was subjected to purification by silica gel flash column chromatography (EtOAc : Hexane – 1 : 49) yielded the desired product as a colourless oil (377 mg, 3.66 mmol, 52%) (TLC: EtOAc – $R_f$ = 0.68). IR (NaCl, 10 mM in $CHCl_3$): 3018, 2982, 2958, 2881, 1685, 1457, 1396, 1132 $cm^{-1}$; $^1$H NMR (400 MHz, $CDCl_3$) δ ppm: 3.69 (s, 3H), 3.39 (t, $J$ = 6.3 Hz, 2H), 3.32 (t, $J$ = 6.3 Hz, 2H), 1.88-1.82 (m, 4H); $^{13}$C NMR (100 MHz, $CDCl_3$) δ ppm: 155.5, 52.1, 46.0, 45.6, 25.6, 24.8; HRMS *m/z* Calcd for $C_6H_{11}NO_2Na$ 152.0687, Found 152.0690.

### -S2.2. Ethyl pyrrolidine-1-carboxylate (2):

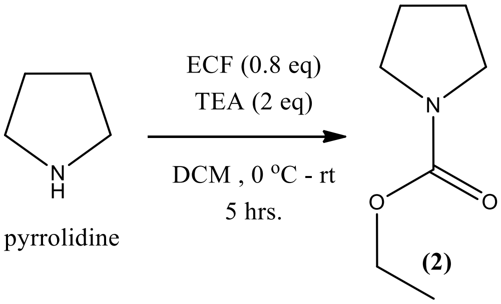


**(2)**

To a cold (0 °C) solution of **pyrrolidine** (500 mg, 7.03 mmol) in 14 mL Dichloromethane (DCM) added Ethyl chloroformate (ECF) (537 µL, 5.62 mmol), triethylamine ($Et_3N$) (1.95 mL, 14.1 mmol) and the mixture was allowed to stir at 0 °C for 10 min, then allowed to stir at rt for 5 h. Removal of solvent resulted in a residue which was dissolved in ethyl acetate (EtOAc) (10 mL), washed with 10 mL water (2 X 5 mL) and 1N HCl solution (2 X 5 mL) and saturated $NaHCO_3$ solution (2 X 5 mL) and dried over anhydrous sodium sulphate ($Na_2SO_4$), and the organic layer was concentrated to get a residue which was subjected to purification by silica gel flash column chromatography (EtOAc : Hexane – 2 : 48) yielded the desired product as a colourless oil (423 mg, 3.73 mmol, 53%) (TLC: EtOAc – $R_f$ = 0.69). IR (NaCl, 10 mM in $CHCl_3$): 3016, 2982, 2880, 1679.1, 1436, 1384, 1130, 1109 $cm^{-1}$; $^1$H NMR (400 MHz, $CDCl_3$) δ ppm: 4.13 (q, $J$ = 7.1 Hz, 2H), 3.38 (t, $J$ = 5.9 Hz, 2H), 3.33 (t, $J$ = 5.9 Hz, 2H), 1.88-1.82 (m, 4H), 1.26 (t, $J$ = 7Hz, 3H); $^{13}$C NMR (100 MHz, $CDCl_3$) δ ppm: 155.0, 60.5, 45.8, 45.4, 25.5, 24.7, 14.6; HRMS *m/z* Calcd for $C_7H_{13}NO_2Na$ 166.0844, Found 166.0841.

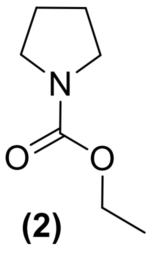


(2)

**-S2.3. Isopropyl pyrrolidine-1-carboxylate (3):**

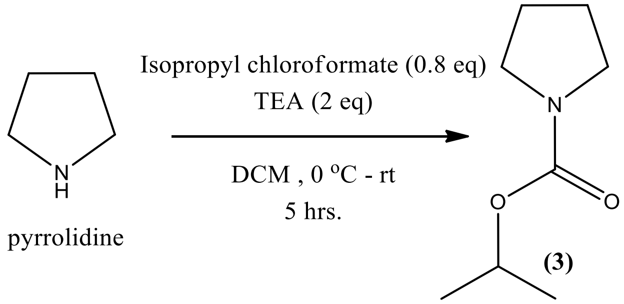


To a cold (0 °C) solution of **pyrrolidine** (500 mg, 7.03 mmol) in 14 mL Dichloromethane (DCM) added Isopropyl chloroformate (7 mL, 5.62 mmol) (1M in toluene), triethylamine ($Et_3N$) (1.95 mL, 14.1 mmol) and the mixture was allowed to stir at 0 °C for 10 min, then allowed to stir at rt for 5 h. Removal of solvent resulted in a residue which was dissolved in ethyl acetate (EtOAc) (10 mL), washed with 10 mL water (2 X 5 mL) and 1N HCl solution (2 X 5 mL) and saturated $NaHCO_3$ solution (2 X 5 mL) and dried over anhydrous sodium sulphate ($Na_2SO_4$), and the organic layer was concentrated to get a residue which was subjected to purification by silica gel flash column chromatography (EtOAc : Hexane – 2 : 48) yielded the

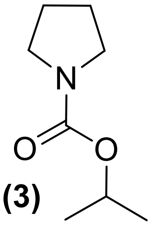


(3)

desired product as a colourless oil (564 mg, 3.66 mmol, 52%) (TLC: EtOAc:Hexane (1:1) – $R_f$ = 0.58). IR (NaCl, 10 mM in $CHCl_3$): 3016, 2982, 2879, 1673.7, 1429, 1215, 1111, 908 $cm^{-1}$; $^1H$ NMR (400 MHz, $CDCl_3$) δ ppm: 4.96-4.87 (m, 1H), 3.37 (t, $J$ = 6.2 Hz, 2H), 3.31 (t, $J$ = 6.3 Hz, 2H), 1.87-1.82 (m, 4H), 1.23 (d, $J$ = 6.2 Hz, 6H); $^{13}C$ NMR (100 MHz, $CDCl_3$) δ ppm: 154.7, 67.6, 45.7, 45.4, 25.5, 24.7, 22.1; HRMS *m/z* Calcd for $C_8H_{15}NO_2Na$ 180.1000, Found 180.1000.

**-S2.4. *tert*-Butyl pyrrolidine-1-carboxylate (4):**

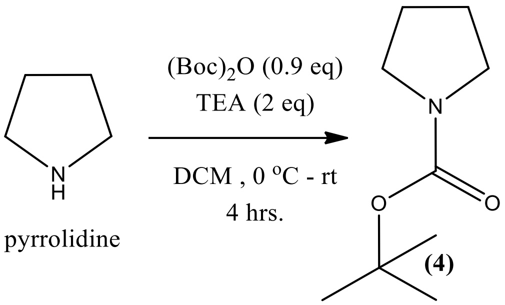


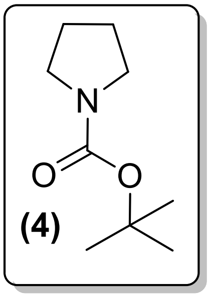


To a cold (0 °C) solution of **pyrrolidine** (500 mg, 7.03 mmol) in 14 mL Dichloromethane (DCM) added Di-tert-butyl dicarbonate ($(Boc)_2O$) (1380 mg, 6.33 mmol), triethylamine ($Et_3N$) (1.95 mL, 14.1 mmol) and the mixture was allowed to stir at 0 °C for 10 min, then allowed to stir at rt for 4 h. Removal of solvent resulted in a residue which was dissolved in ethyl acetate (EtOAc) (10 mL), washed with 10 mL water (2 X 5 mL) and 1N HCl solution (2 X 5 mL) and saturated $NaHCO_3$ solution (2 X 5 mL) and dried over anhydrous sodium sulphate ($Na_2SO_4$), and the organic layer was concentrated to get a residue which was subjected to purification by silica gel flash column chromatography (EtOAc : Hexane – 1 : 49) yielded the desired product as a colourless oil (650 mg, 3.8 mmol, 54%) (TLC: EtOAc:Hexane (1:1) – $R_f$ = 0.68). IR (NaCl, 10 mM in $CHCl_3$): 3006, 2980, 2880, 1683.4, 1417, 1259, 1165, 758, 688 $cm^{-1}$; $^1H$ NMR (400 MHz, $CDCl_3$) δ ppm: 3.35-3.31 (m, 2H), 3.29-3.26 (m, 2H), 1.85-1.81 (m, 4H), 1.46 (s, 9H); $^{13}C$ NMR (100 MHz, $CDCl_3$) δ ppm: 154.5, 78.7, 45.8, 45.5, 28.4, 25.6, 24.8; HRMS *m/z* Calcd for $C_9H_{17}NO_2Na$ 194.1157, Found 194.1152.

**-S3. $^{1}H$ NMR and $^{13}C$ NMR Spectram of carbamates (1-4).**
**-S3.1. Figure 1.** $^{1}H$ NMR of **1** in $CDCl_3$ (400 MHz, 10 mmol).

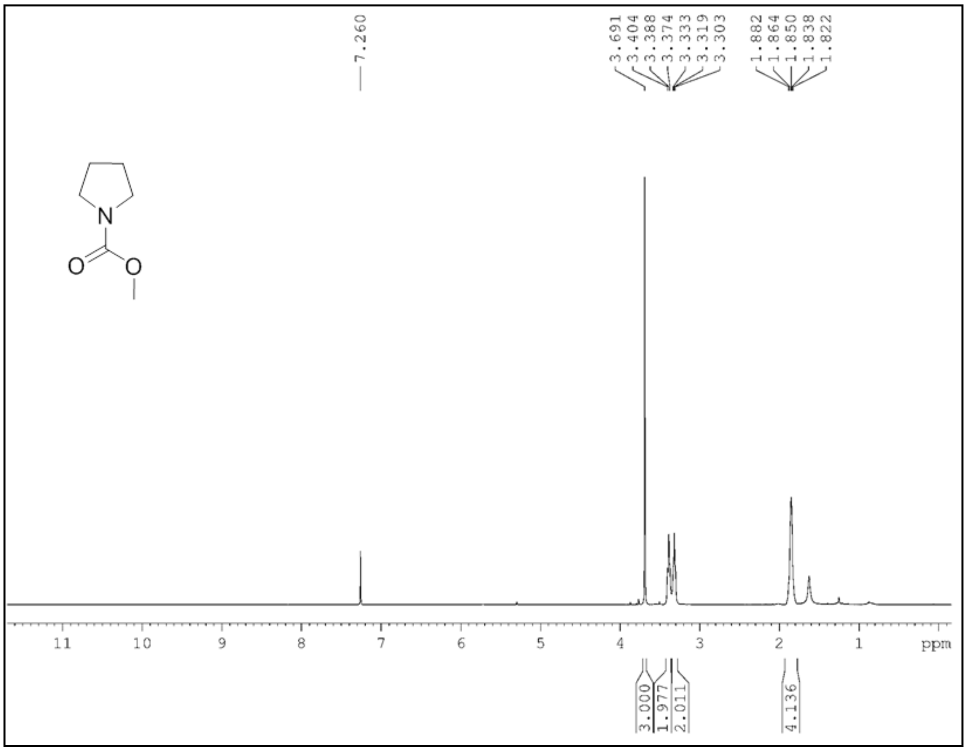


**-S3.2. Figure 2.** $^{13}C$ NMR of **1** in $CDCl_3$ (100 MHz, 60 mmol).

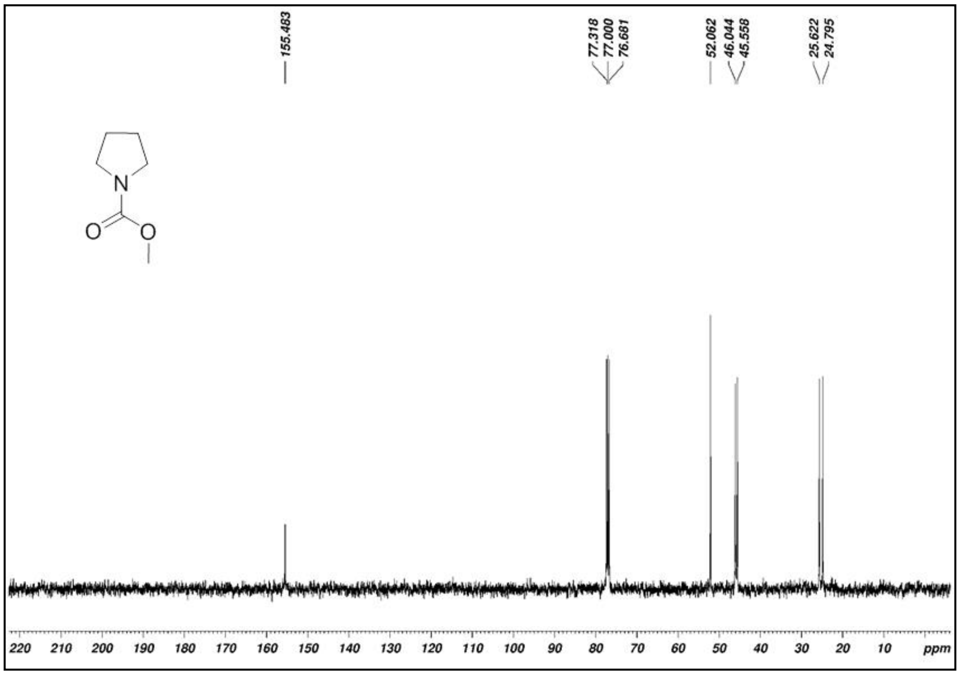

-**S3.3. Figure 3.** $^{1}$H NMR of **2** in $CDCl_3$ (400 MHz, 10 mmol).

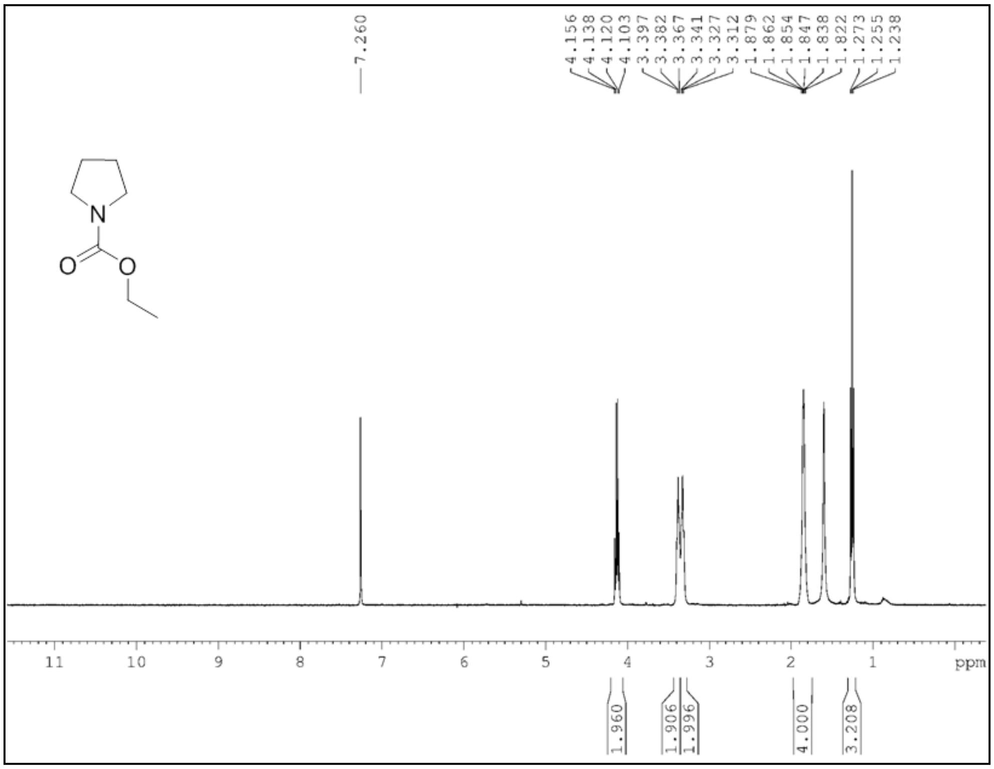


-**S3.4. Figure 4.** $^{13}$C NMR of **2** in $CDCl_3$ (100 MHz, 60 mmol).

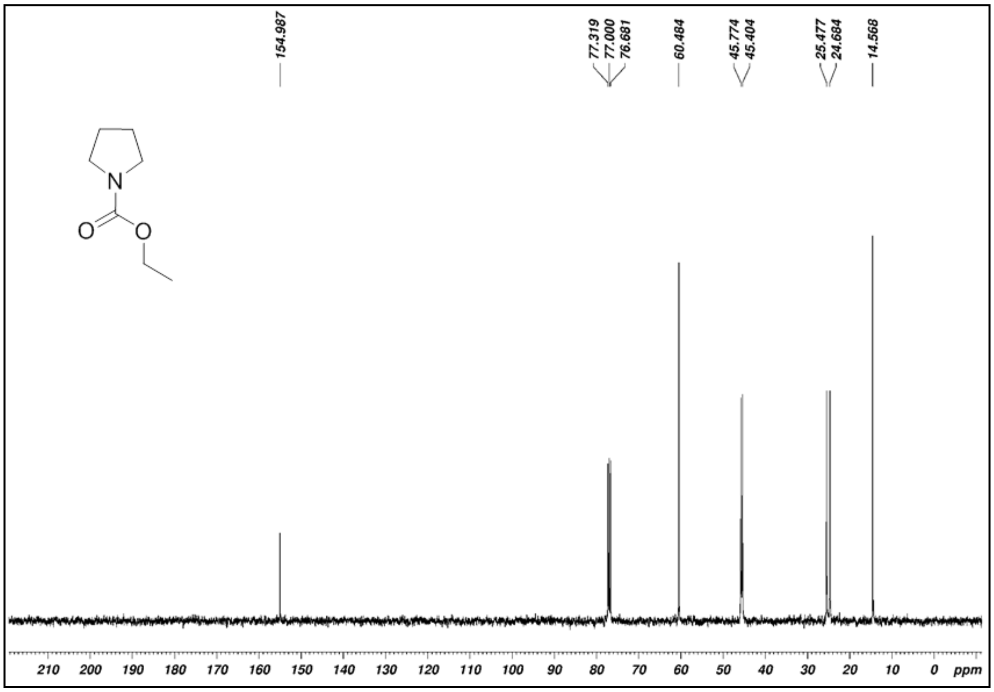

**-S3.5. Figure 5.** $^{1}$H NMR of **3** in $CDCl_3$ (400 MHz, 10 mmol).

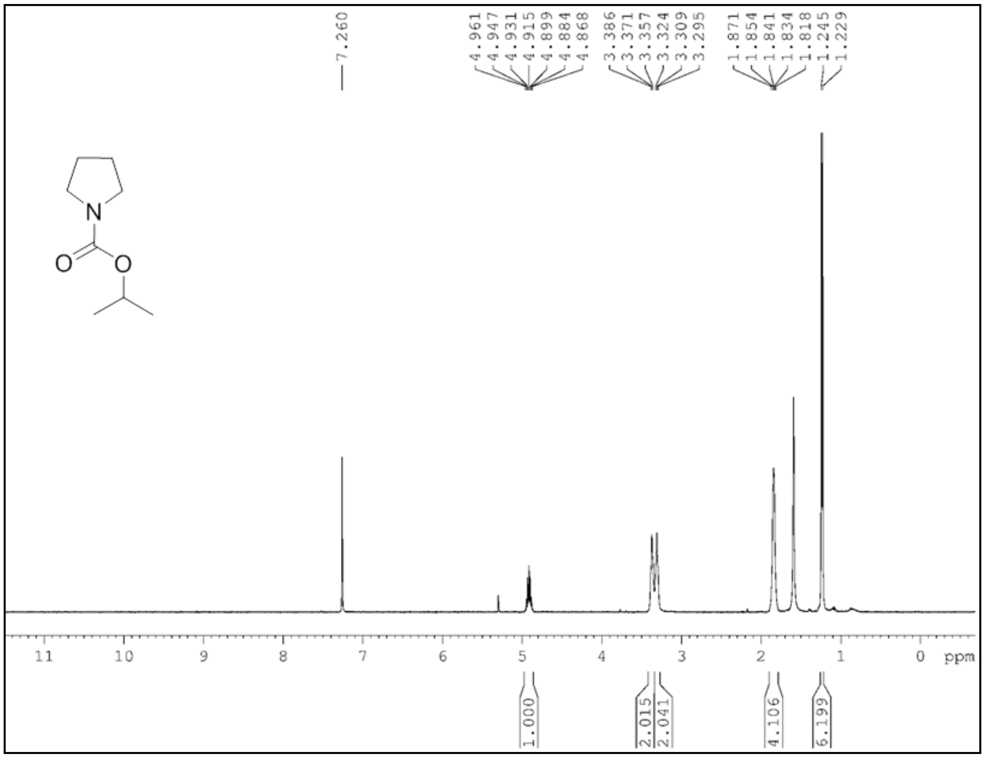


**-S3.6. Figure 6.** $^{13}$C NMR of **3** in $CDCl_3$ (100 MHz, 60 mmol).

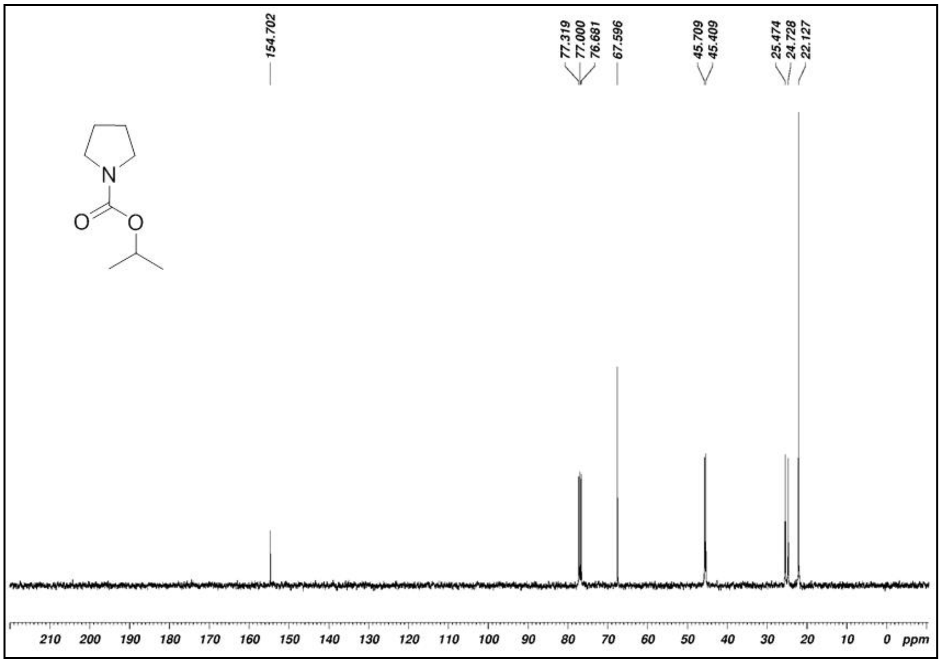

**-S3.7. Figure 7.** $^{1}$H NMR of **4** in $CDCl_3$ (400 MHz, 10 mmol).

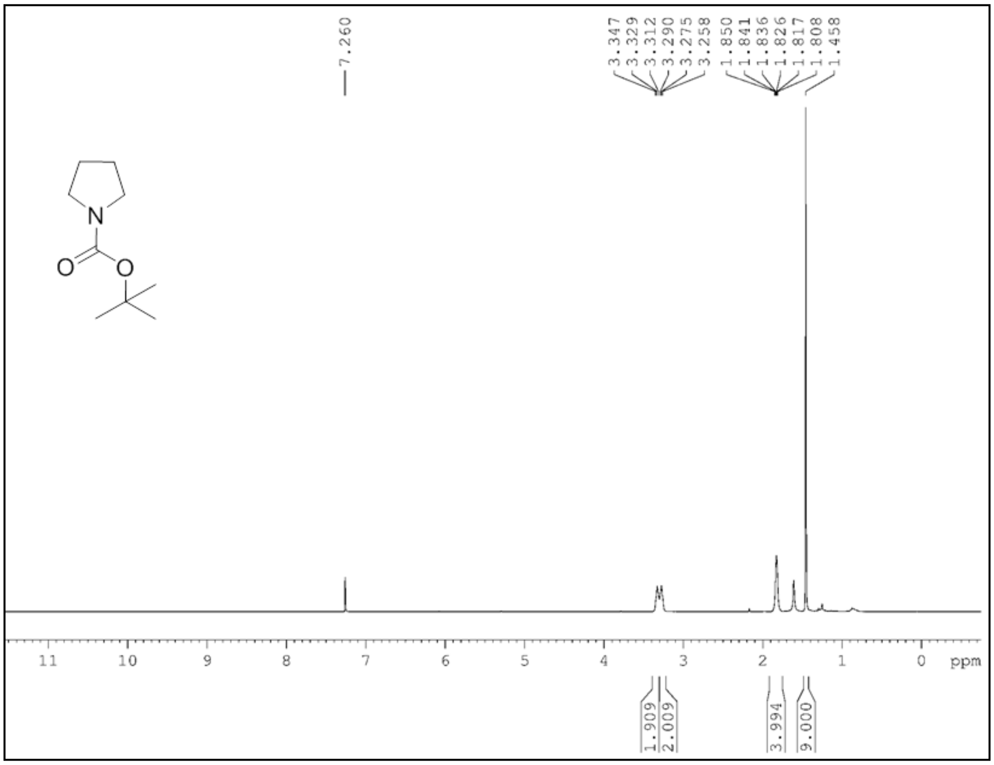


**-S3.8. Figure 8.** $^{13}$C NMR of **4** in $CDCl_3$ (100 MHz, 60 mmol).

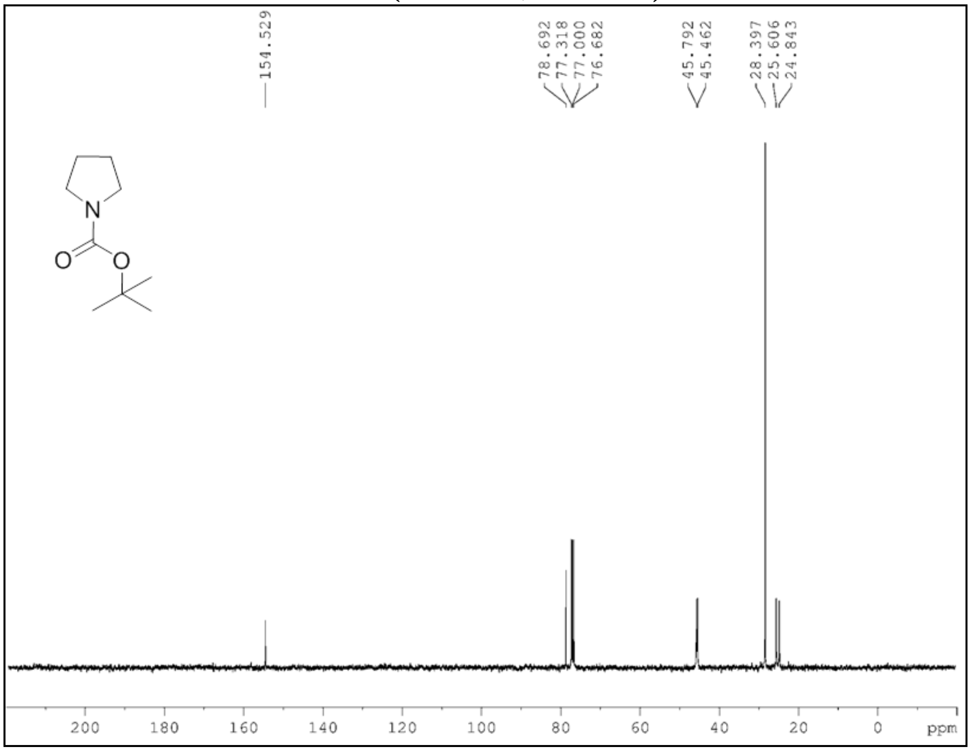

**-S4. Figure 9. Stacked FT-IR spectra (C=O stretching frequency region) of 1 to 4 ($CHCl_3$, 10 mmol)**

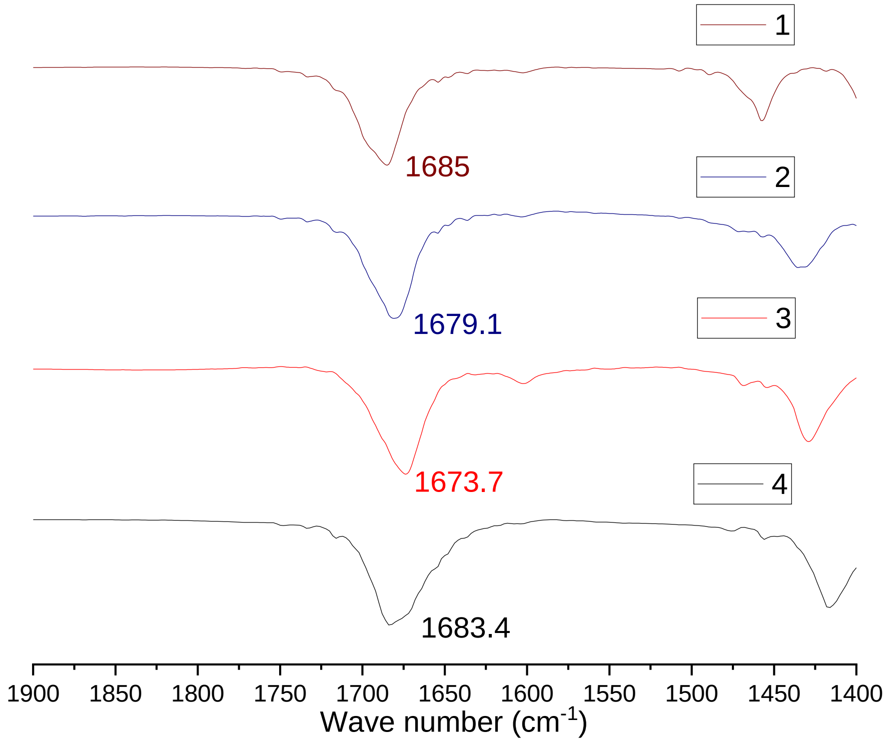


**-S5.a. Table. 1 Comparison of relevant $^{1}H$, $^{13}C$ NMR ($CDCl_3$) and FT-IR spectral data ($CHCl_3$) of homologous Pyrrolidine carbamates (1-4).**

a)

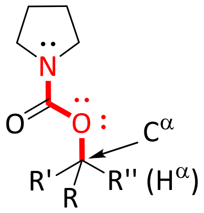


| | R | R' | R'' |
|---|---|---|---|
| **1** | H | H | H |
| **2** | Me | H | H |
| **3** | Me | Me | H |
| **4** | Me | Me | Me |

b)

| | R | R' | R'' | $^{13}C$ NMR $C^{\alpha}$ of O-C δ ppm | C=O FTIR υ $cm^{-1}$ | $^{13}C$ NMR O=C δ ppm | $^{1}H$ NMR $OC^{\alpha}$-H δ ppm | $^{13}C$ NMR $C^{\beta}$ of O-C-$C^{\beta}$ δ ppm | $^{1}H$ NMR O-C-$C^{\beta}$-H δ ppm |
|---|---|---|---|---|---|---|---|---|---|
| **1** | H | H | H | 52.1 | 1685.0 | 155.5 | 3.69 | NA | NA |
| **2** | Me | H | H | 60.5 | 1679.1 | 155.0 | 4.13 | 14.6 | 1.26 |
| **3** | Me | Me | H | 67.6 | 1673.7 | 154.7 | 4.92 | 22.1 | 1.23 |
| **4** | Me | Me | Me | 78.7 | 1683.4 | 154.5 | NA | 28.4 | 1.46 |

**-S5.b. Table. 2 Comparison of relevant $^{1}H$, $^{13}C$ NMR ($CDCl_3$) and FT-IR spectral data ($CHCl_3$) of homologous N-phenyl carbamates (5-8)[1-3].**

a)

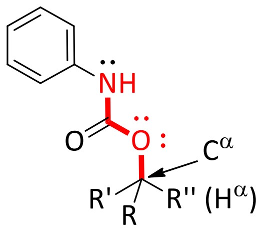


| | R | R' | R'' |
|---|---|---|---|
| **5** | H | H | H |
| **6** | Me | H | H |
| **7** | Me | Me | H |
| **8** | Me | Me | Me |

b)

| | R | R' | R'' | $^{13}$C NMR $C^{\alpha}$ of O-C δ ppm | C=O FTIR υ $cm^{-1}$ | $^{13}$C NMR O=C δ ppm | $^{1}$H NMR $OC^{\alpha}$-H δ ppm | $^{13}$C NMR $C^{\beta}$ of O-C-$C^{\beta}$ δ ppm | $^{1}$H NMR O-C-$C^{\beta}$-H δ ppm | Ref |
|---|---|---|---|---|---|---|---|---|---|---|
| **5** | H | H | H | 52.3 | 1705 | 154.1 | 3.78 | NA | NA | 1 |
| **6** | Me | H | H | 61.1 | 1701 | 153.6 | 4.23 | 14.5 | 1.31 | 1 |
| **7** | Me | Me | H | 68.7 | - | 153.7 | 5.05 | 22.1 | 1.30 | 2 |
| **8** | Me | Me | Me | 80.4 | 1689 | 152.7 | NA | 28.3 | 1.51 | 3 |

**-S5.c. Table. 3 Comparison of relevant $^{1}H$, $^{13}C$ NMR ($CDCl_3$) and FT-IR spectral data of homologous Alkyl acetates (9-12).**

a)

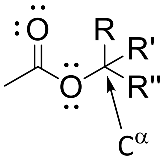


| | R | R' | R'' |
|---|---|---|---|
| **9** | H | H | H |
| **10** | Me | H | H |
| **11** | Me | Me | H |
| **12** | Me | Me | Me |

b)

| | R | R' | R'' | $^{13}$C NMR $C^{\alpha}$ of O-C δ ppm | C=O FTIR υ $cm^{-1}$ | $^{13}$C NMR O=C δ ppm | $^{1}$H NMR $OC^{\alpha}$-H δ ppm | $^{13}$C NMR $C^{\beta}$ of O-C-$C^{\beta}$ δ ppm | $^{1}$H NMR O-C-$C^{\beta}$-H δ ppm |
|---|---|---|---|---|---|---|---|---|---|
| **9** | H | H | H | 51.6 | 1748 | 171.5 | 3.67 | NA | NA |
| **10** | H | H | Me | 60.5 | 1740 | 171.4 | 4.12 | 14.2 | 1.25 |
| **11** | H | Me | Me | 67.6 | 1736 | 170.6 | 4.99 | 21.8 | 1.23 |
| **12** | Me | Me | Me | 80.1 | 1738 | 170.4 | NA | 28.1 | 1.45 |

**-S5.d. Table. 4 Comparison of relevant $^1H$, $^{13}C$ NMR ($CDCl_3$) and FT-IR spectral data of homologous Alkyl benzoates (13-16)[4-9].**

a)

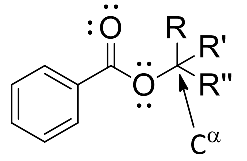

| | R | R' | R'' |
|---|---|---|---|
| **13** | H | H | H |
| **14** | Me | H | H |
| **15** | Me | Me | H |
| **16** | Me | Me | Me |

b)

| | R | R' | R'' | $^{13}C$ NMR $C^{\alpha}$ of O-C $\delta$ ppm | C=O FTIR $\upsilon$ $cm^{-1}$ | $^{13}C$ NMR O=C $\delta$ ppm | $^1H$ NMR $OC^{\alpha}$-H $\delta$ ppm | $^{13}C$ NMR $C^{\beta}$ of O-C-$C^{\beta}$ $\delta$ ppm | $^1H$ NMR O-C-$C^{\beta}$-H $\delta$ ppm | Ref |
|---|---|---|---|---|---|---|---|---|---|---|
| **13** | H | H | H | 51.5 | 1725 | 165.9 | 3.92 | NA | NA | 4,5,6 |
| **14** | H | H | Me | 60.8 | 1720 | 166.5 | 4.33 | 14.3 | 1.33 | 7,8 |
| **15** | H | Me | Me | 68.3 | 1716 | 166.1 | 5.24 | 21.9 | 1.35 | 6,9 |
| **16** | Me | Me | Me | 80.9 | - | 165.8 | NA | 28.2 | 1.58 | 6 |

**-S5.e. Table. 5 Comparison of relevant $^1H$, $^{13}C$ NMR ($CDCl_3$) and FT-IR spectral data of homologous alcohols (17-20)[10, 11].**

a)

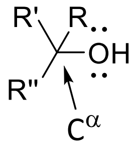

| | R | R' | R'' |
|---|---|---|---|
| **17** | H | H | H |
| **18** | Me | H | H |
| **19** | Me | Me | H |
| **20** | Me | Me | Me |

b)

| | R | R' | R'' | $^{13}C$ NMR $C^{\alpha}$ of O-C $\delta$ ppm | $^1H$ NMR $OC^{\alpha}$-H $\delta$ ppm | $^{13}C$ NMR $C^{\beta}$ of O-C-$C^{\beta}$ $\delta$ ppm | $^1H$ NMR O-C-$C^{\beta}$-H $\delta$ ppm |
|---|---|---|---|---|---|---|---|
| **17** | H | H | H | 50.41 | 3.48 | NA | NA |
| **18** | H | H | Me | 58.3 | 3.72 | 18.4 | 1.24 |
| **19** | H | Me | Me | 64.50 | 4.03 | 25.1 | 1.20 |
| **20** | Me | Me | Me | 69.15 | NA | 31.25 | 1.27 |

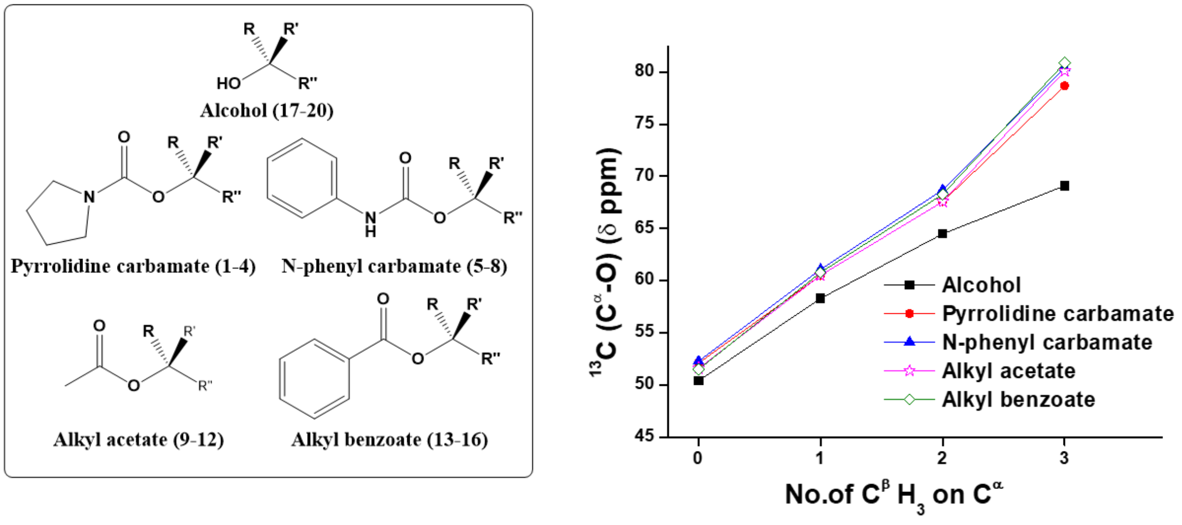


**-S6.a. Figure 10.** The incremental downfield shift in $^{13}C^{\alpha}$ nuclear signals as the number of methyl substituents on $C^{\alpha}$-O increase in homologous alcohols. The steeper incremental shifts in corresponding pyrrolidine carbamates and acetates. Note the similarity in shifts between carbamates and esters. Such significant shifts indicate hyperconjugative resonance stabilization of the positive charge polarization at $C^{\alpha}$ of alcohol. These are further highlighted in the difference chemical shift plot in the next figure.

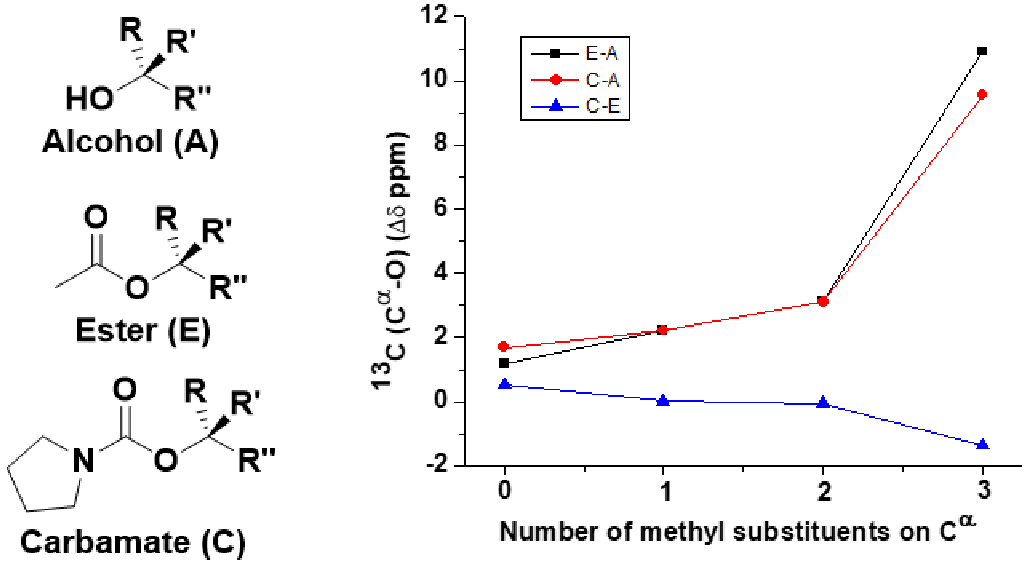


**-S6.b. Figure 11.** The plot of the difference in $C^{\alpha}$ chemical shifts between Pyrrolidine carbamate (**C; 1-4**), Alkyl acetate (**E; 9-12**) and Alcohol (**A; 17-20**) against the increasing number of methyl substituents on $C^{\alpha}$ of alcohol. Similar patterns were observed for N-phenyl carbamate (**5-8**) and Alkyl benzoate (**13-16**).

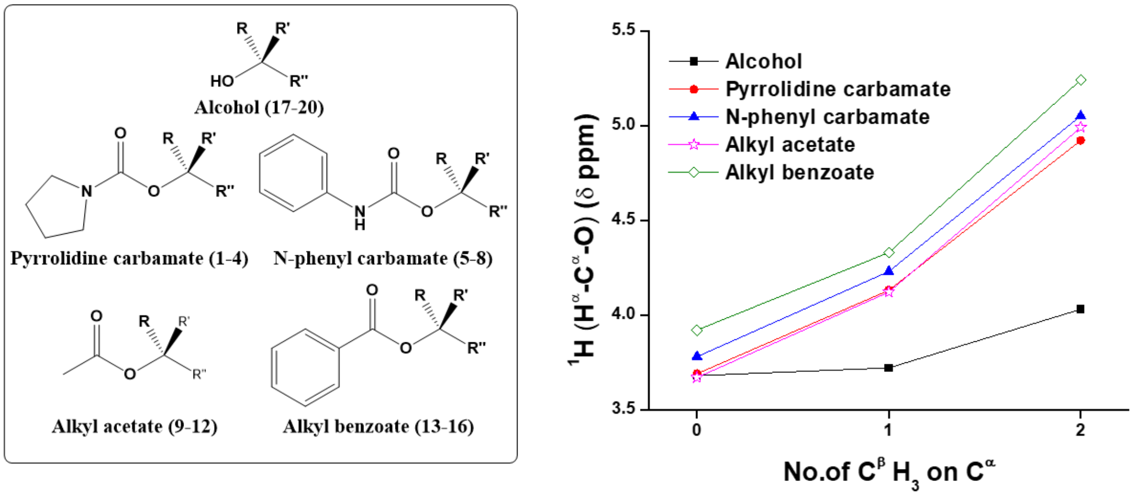


**-S6.c. Figure 12.** The incremental downfield shift in $^{1}H^{\alpha}$ nuclear signals as the number of methyl substituents on $C^{\alpha}$-O increase in homologous alcohols. The steeper incremental shifts in corresponding pyrrolidine carbamates and acetates. Note the similarity in shifts between carbamates and esters. Such significant shifts indicate hyperconjugative resonance stabilization of the positive charge polarization at $C^{\alpha}$ of alcohol. These are further highlighted in the difference chemical shift plot in the next figure.

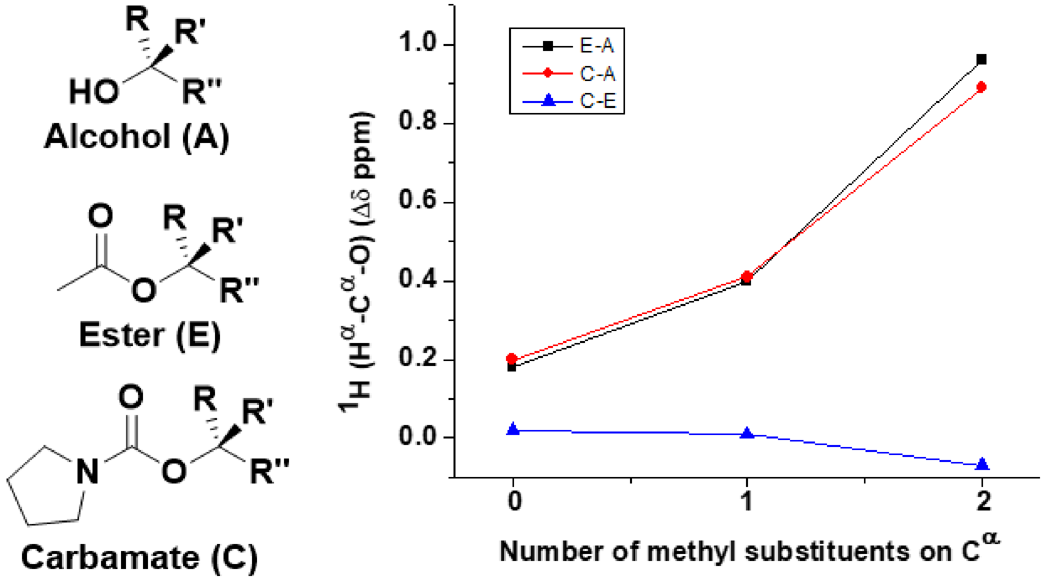


**-S6.d. Figure 13.** The plot of the difference in $H^{\alpha}$ chemical shifts between Pyrrolidine carbamate (**C; 1-4**), Alkyl acetate (**E; 9-12**) and Alcohol (**A; 17-20**) against the increasing number of methyl substituents on $C^{\alpha}$ of alcohol**.** Similar patterns were observed for N-phenyl carbamate (**5-8**) and Alkyl benzoate (**13-16**).

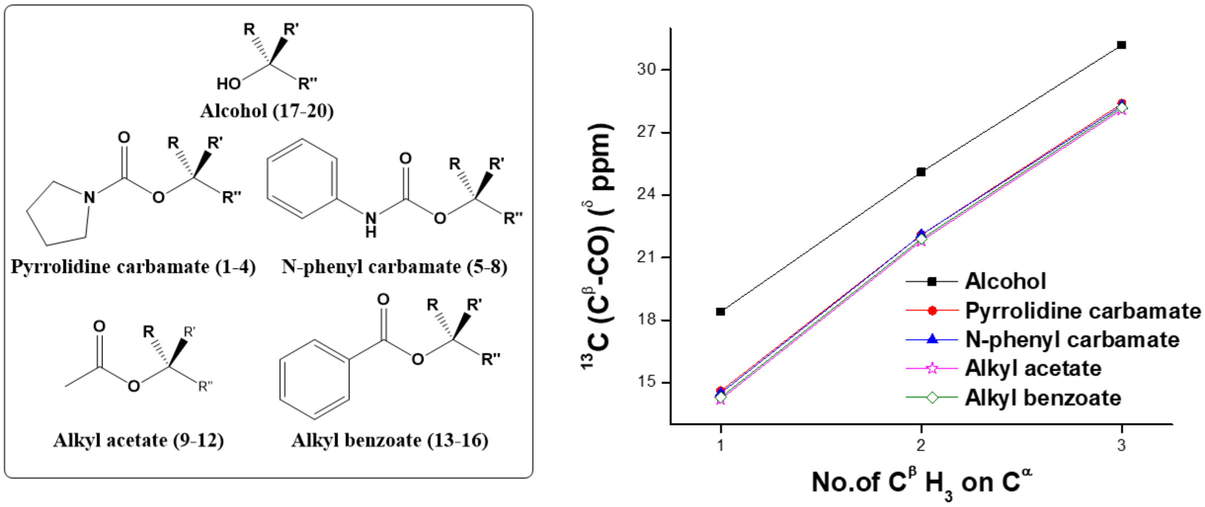


**-S6.e. Figure 14.** The incremental downfield shift in $^{13}C^{\beta}$ nuclear signals as the number of methyl substituents on $C^{\alpha}$-O increase in homologous alcohols. The similar incremental shifts in corresponding pyrrolidine carbamates and acetates are more upfield shifted compared to the corresponding alcohols. Such significant upfield shifts substantiate the back donation of electronic charge from carbonyl oxygen to the $C^{\alpha}$ of alcohol (O…$C^{\alpha}$). Note the similarity in shifts between carbamates and esters.

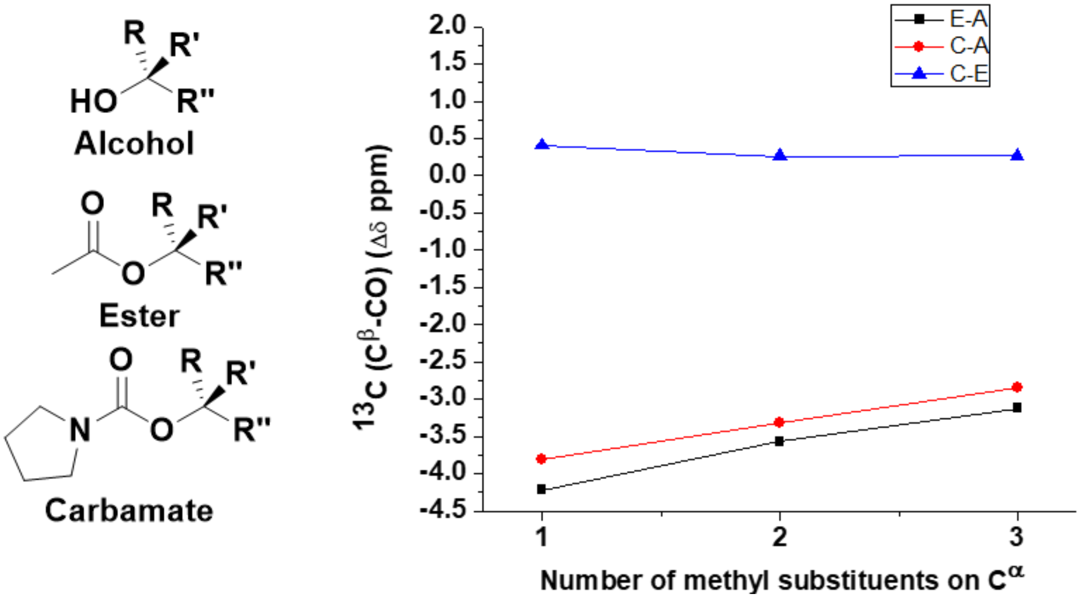


**-S6.f. Figure 15.** Plot of difference in $^{13}C$ NMR chemical shifts of $C^{\beta}$ between Pyrrolidine carbamate (**C; 1-4**), Alkyl acetate (**E; 9-12**) and Alcohol (**A; 17-20**) against the increasing number of methyl substituents on $C^{\alpha}$ of alcohol**.** Note that the $C^{\beta}$ in esters are slightly more upfield shifted compared to those in carbamates, indicating greater electronic charge on their carbonyl oxygen. Similar patterns were observed for N-phenyl carbamate (**5-8**) and Alkyl benzoate (**13-16**).

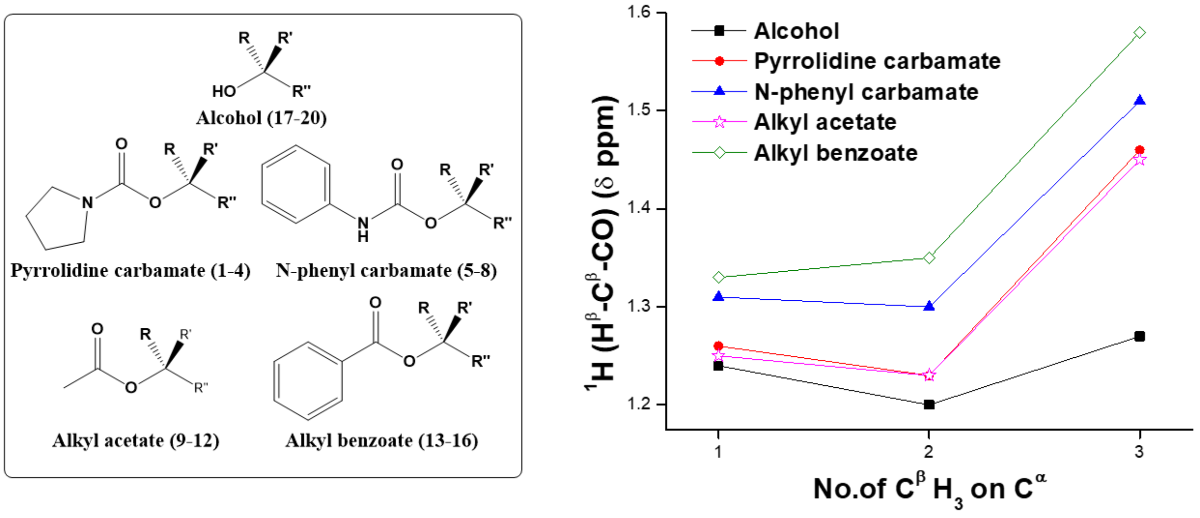


**-S6.g. Figure 16.** The plot of ¹H NMR chemical shift of $H^{\beta}$ of alcohol group in esters, carbamates and alcohols against the number of methyl substituents on $C^{\alpha}$.

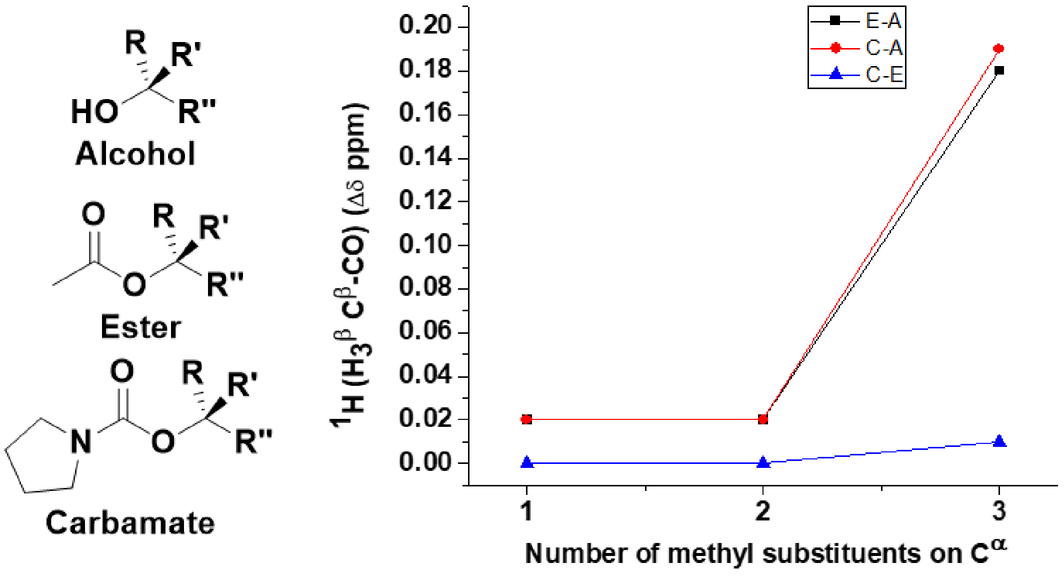


**-S6.h. Figure 17.** Plot of difference in ¹H NMR chemical shifts of $H^{\beta}$ between Pyrrolidine carbamate (**C; 1-4**), Alkyl acetate (**E; 9-12**) and Alcohol (**A; 17-20**) against the increasing number of methyl substituents on $C^{\alpha}$ of alcohol**.** Similar patterns were observed for N-phenyl carbamate (**5-8**) and Alkyl benzoate (**13-16**).

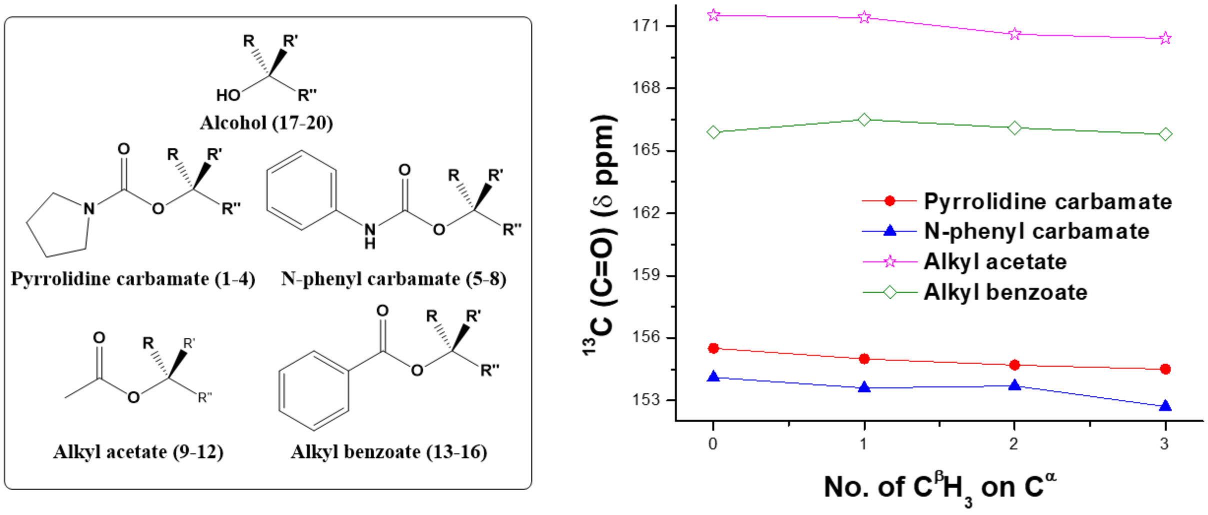


**-S6.i. Figure 18.** The downfield shift in [13]C nuclear signals of C=O in carbamates compared to acetates of homologous alcohols. Both of them are relatively unperturbed by increasing number of methyl substituents on $C^{\alpha}$ of alcohol. Note that the trend is uniform and non-incremental between the two.

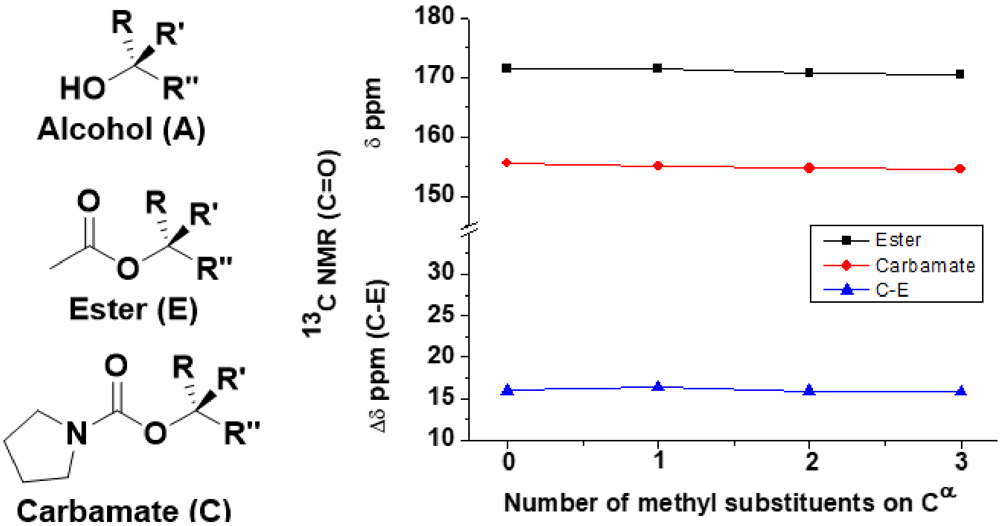


**-S6.j. Figure 19.** The correlation plots of the [13]C nuclear signals of carbonyls in Pyrrolidine carbamate (**C; 1-4**), Alkyl acetate (**E; 9-12**) of homologous alcohols versus the number of methyl substituents at $C^{\alpha}$. The difference plot for C-E is also shown in blue, highlighting their non-dependence on the number of methyl substituents at $C^{\alpha}$. Similar patterns were observed for N-phenyl carbamate (**5-8**) and Alkyl benzoate (**13-16**).

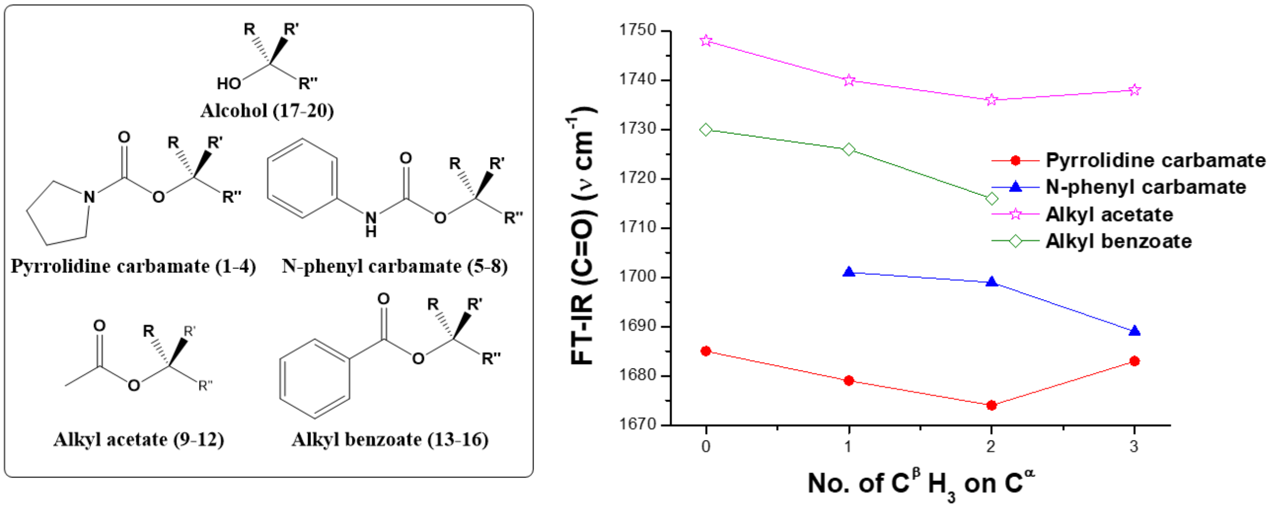


**-S6.k. Figure 20.** The decrease in C=O stretching frequencies in carbamates and Ester of homologous alcohols, with increasing number of methyl substituents on C$^{\alpha}$ of alcohol. Note that the trend is uniform rather than incremental between the two.

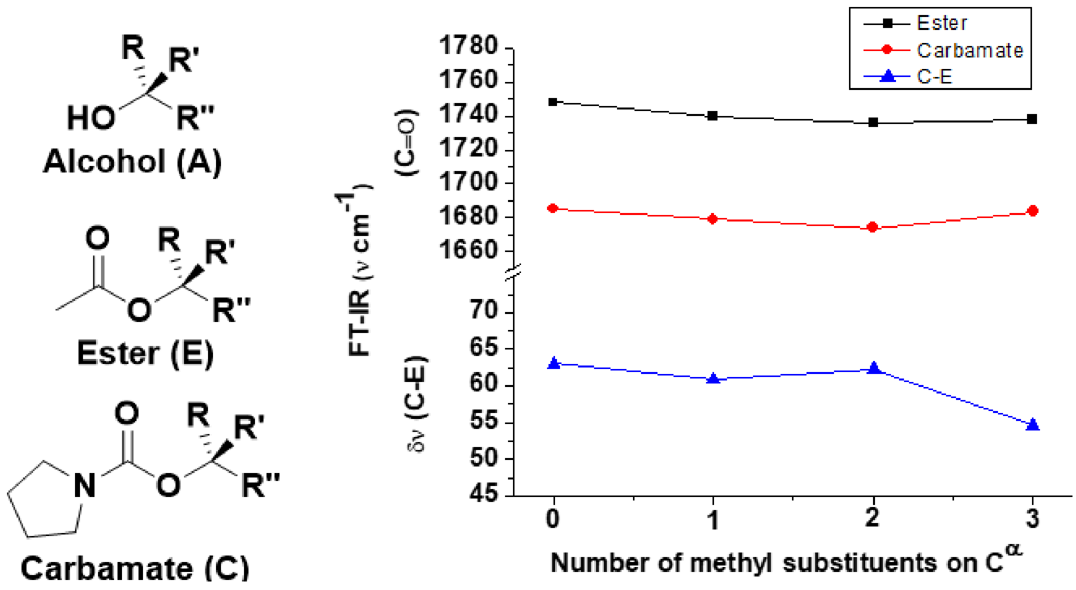


**-S6.l. Figure 21.** The correlation plots of the C=O stretching frequency in FT-IR spectra of Pyrrolidine carbamate (**C; 1-4**), Alkyl acetate (**E; 9-12**) of homologous alcohols versus the number of methyl substituents at C$^{\alpha}$. The difference plot for C-E is also shown in blue. Similar patterns were observed for N-phenyl carbamate (**5-8**) and Alkyl benzoate (**13-16**).

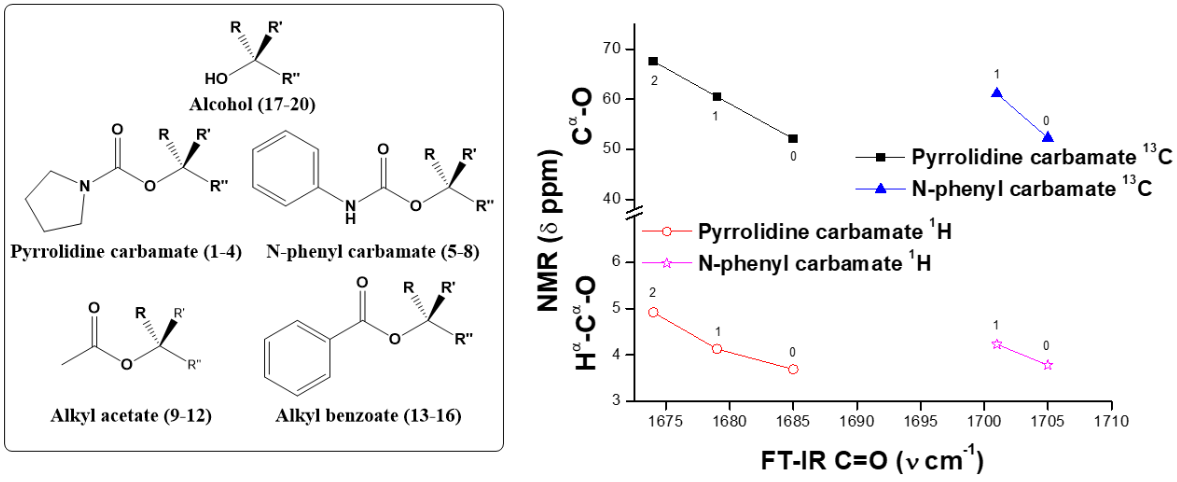


**-S6.m. Figure 22.** The combined correlation plots of the Cα and Hα nuclear chemical shifts of carbamates versus the FT-IR stretching frequencies of the corresponding homologous alcohols. Note the negative slopes and hence the inverse correlation between the NMR and FT-IR data, indicating the influence of the number of substituents at Cα on the bond order of C=O and hence the electronic charge at the carbonyl oxygen.

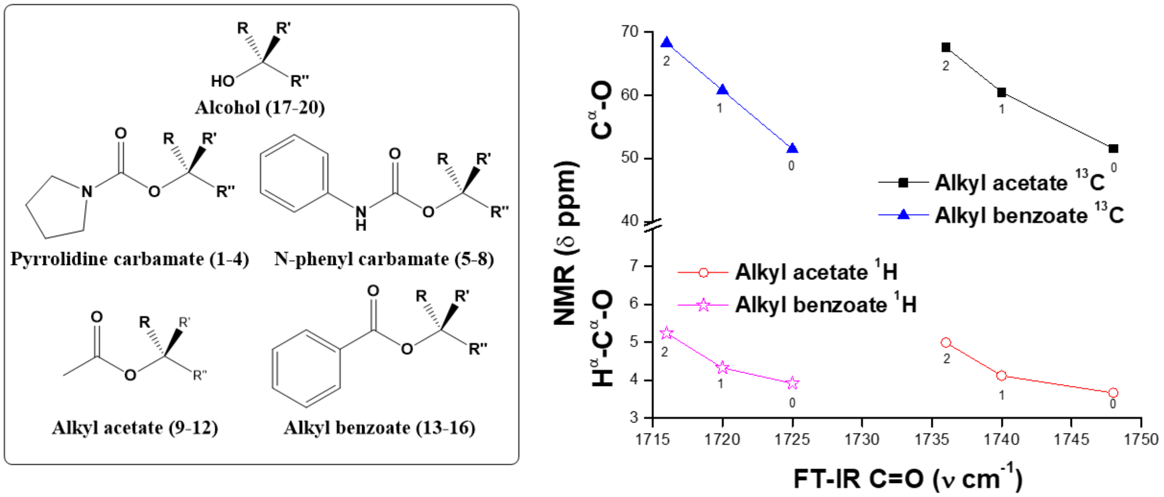


**S6.n. Figure 23.** The combined correlation plots of the Cα and Hα nuclear chemical shifts of esters versus the FT-IR stretching frequencies of the corresponding homologous alcohols. Note the negative slopes and hence the inverse correlation between the NMR and FT-IR data, indicating the influence of the number of substituents at Cα on the bond order of C=O and hence the electronic charge at the carbonyl oxygen.

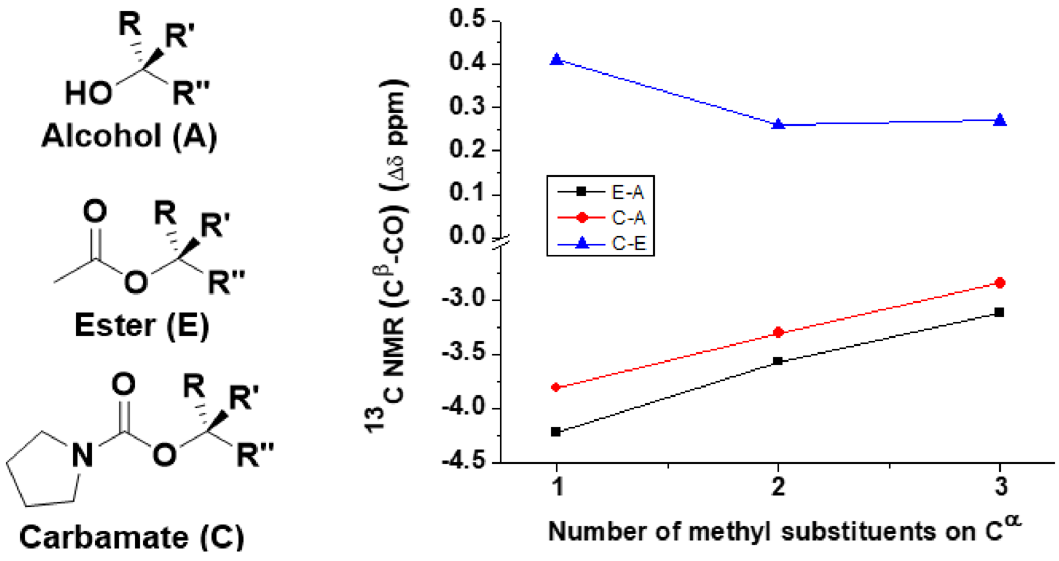


**-S6.o. Figure 24.** The correlation plots of the difference in $C^{\beta}$ chemical shifts between Pyrrolidine carbamate (**C; 1-4**), Alkyl acetate (**E; 9-12**) and Alcohol (**A; 17-20**) against the increasing number of methyl substituents on $C^{\alpha}$ of alcohol. Note the reversal in trend – where the $C^{\beta}$ of carbamates and esters are upfield shifted compared to those in alcohols, in contrast to their $C^{\alpha}$ which are downfield shifted. Similar patterns were observed for N-phenyl carbamate (**5-8**) and Alkyl benzoate (**13-16**).

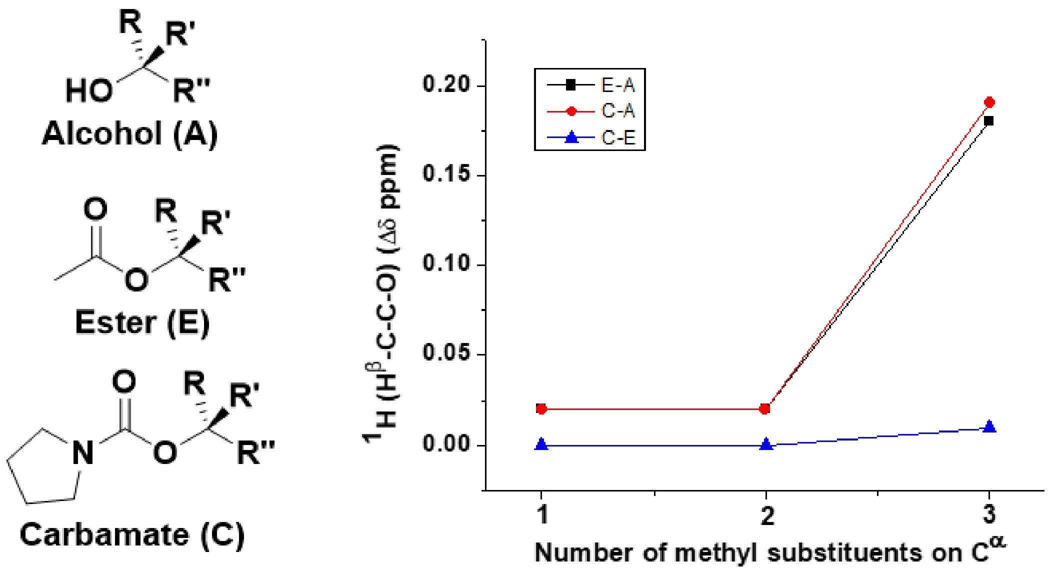


**-S6.p. Figure 25.** The correlation plots of the difference in $H^{\beta}$ chemical shifts between Pyrrolidine carbamate (**C; 1-4**), Alkyl acetate (**E; 9-12**) and Alcohol (**A; 17-20**) against the increasing number of methyl substituents on $C^{\alpha}$ of alcohol. Note the reversal in trend – where the $H^{\beta}$ of carbamates and esters are upfield shifted compared to those in alcohols, in contrast to their $H^{\alpha}$ which are

downfield shifted. Similar patterns were observed for N-phenyl carbamate (**5-8**) and Alkyl benzoate (**13-16**).

# -S7. Table 6. Structural parameters at the R-O-C=O register (s-trans conformation) in carbamates and esters and from corresponding crystal structures[12-30].

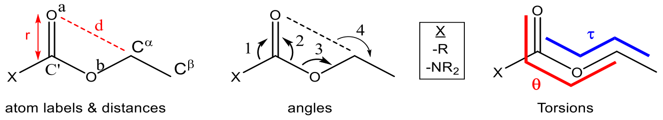


| | Molecule | CCDC No | distances (Å) r | distances (Å) d | angles (degrees) 1 | angles (degrees) 2 | angles (degrees) 3 | angles (degrees) 4 | Torsions (degrees) θ | Torsions (degrees) τ | Ref |
|---|---|---|---|---|---|---|---|---|---|---|---|
| carbamates | 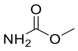 | 1154688 | 1.23 | 2.63 | 124.2 | 122.9 | 115.2 | 164.4 | 1.32 | 171.45 | 12 |
| | 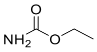 | 1148417 | 1.22 | 2.66 | 124.9 | 123.4 | 115.6 | 162.6 | 1.04 | -178.08 | 13 |
| | 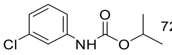 | 723495 | 1.21 | 2.72 | 126.4 | 124.7 | 117.3 | 146.2 | -3.84 | 150.34 | 14 |
| | 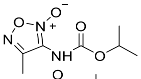 | 1238012 | 1.21 | 2.75 | 123.9 | 126.4 | 117.3 | 149.4 | 7.12 | -156.74 | 15 |
| | 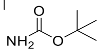 | 1110475 | 1.22 | 2.81 | 124.4 | 125.0 | 121.2 | 156.1 | 2.75 | 179.06 | 16 |
| | 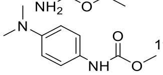 | 1168367 | 1.24 | 2.70 | 124.9 | 123.1 | 118.3 | 167.5 | 1.62 | 179.37 | 17 |
| | 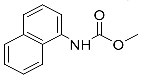 | 1216833 | 1.21 | 2.65 | 125.2 | 123.8 | 115.6 | 170.6 | -0.21 | 179.63 | - |
| | 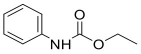 | 1223404 | 1.21 | 2.68 | 126.2 | 123.8 | 116.5 | 165.9 | -0.14 | -161.55 | 18 |
| | 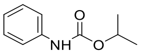 | 1144641 | 1.19 | 2.69 | 122.1 | 130.4 | 113.9 | 132.9 | 3.18 | -143.24 | 19 |
| | 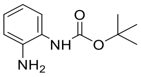 | 785134 | 1.22 | 2.82 | 125.0 | 125.2 | 120.8 | 171.9 | 3.78 | 176.58 | 20 |
| | 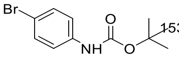 | 1535842 | 1.21 | 2.82 | 124.9 | 125.6 | 121.5 | 172.1 | -3.47 | -175.03 | 21 |
| Esters | 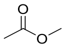 | 1105316 | 1.20 | 2.61 | 125.7 | 122.5 | 114.9 | 153.7 | -1.28 | 178.90 | 22 |
| | 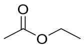 | 904088 | 1.20 | 2.66 | 125.3 | 123.0 | 116.4 | 164.4 | -0.31 | 179.14 | 23 |
| | 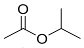 | 794904 | 1.21 | 2.70 | 124.9 | 123.7 | 117.3 | 150.2 | 5.74 | -155.61 | 24 |
| | 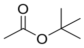 | 748622 | 1.20 | 2.81 | 124.5 | 125.3 | 121.0 | 153.1 | 2.18 | 172.33 | 25 |
| | 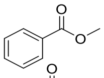 | 844082 | 1.21 | 2.65 | 124.3 | 123.6 | 115.9 | 165.0 | 2.22 | 179.58 | 26 |
| | 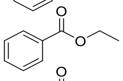 | 144210 | 1.20 | 2.64 | 124.7 | 122.2 | 115.8 | 168.9 | -1.21 | 177.96 | 27 |
| | 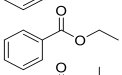 | 160615 | 1.21 | 2.63 | 126.6 | 121.9 | 115.0 | 156.8 | -2.23 | -174.54 | 28 |
| | 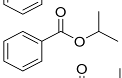 | 1248195 | 1.22 | 2.75 | 123.7 | 124.1 | 119.9 | 144.9 | -1.97 | 160.28 | 29 |
| | 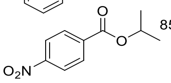 | 850675 | 1.21 | 2.73 | 123.2 | 124.9 | 117.8 | 132.6 | 2.85 | 140.64 | 30 |

angle ∠1 = X-C'=O (degrees)
angle ∠2 = $O^b$-C'=O (degrees)
angle ∠3 = C'-$O^b$-$C^{\alpha}$ (degrees)
angle ∠4 = $O^a$-$C^{\alpha}$-$H^{\alpha}$ (degrees)

r = C=O distance (Å)
d = distance between $O^a$ and $C^{\alpha}$ (Å)
Torsion θ = $O^a$-C'-$O^b$-$C^{\alpha}$ (degrees)
Torsion τ = C'-$O^b$-$C^{\alpha}$-$C^{\beta}$ (degrees)

## References:


1. A. J. Borah and P. Phukan, *Tetrahedron Letters*, 2012, **53**, 3035-3037.
2. R. Ghosh, M. Nethaji and A. G. Samuelson, *Journal of organometallic chemistry*, 2005, **690**, 1282-1293.
3. S. V. Chankeshwara and A. K. Chakraborti, *Tetrahedron letters*, 2006, **47**, 1087-1091 %@ 0040-4039.
4. N. Sundaraganesan and B. D. Joshua, *Spectrochimica Acta Part A: Molecular and Biomolecular Spectroscopy*, 2007, **68**, 771-777 %@ 1386-1425.
5. R. J. Abraham, B. Bardsley, M. Mobli and R. J. Smith, *Magnetic Resonance in Chemistry*, 2005, **43**, 3-15 %@ 0749-1581.
6. S. Pramanik, R. R. Reddy and P. Ghorai, *Organic letters*, 2015, **17**, 1393-1396 %@ 1523-7060.
7. M. Hosseini-Sarvari and E. Sodagar, *Comptes Rendus Chimie*, 2013, **16**, 229-238.
8. J. A. Soderquist, I. Rosado, Y. Marrero and C. Burgos, *ARKIVOC*, 2001, **4**, 12-19.
9. X.-S. Jia, H.-L. Wang, Q. Huang, L.-L. Kong and W.-H. Zhang, *Journal of Chemical Research*, 2006, **2006**, 135-138 %@ 1747-5198.
10. N. R. Babij, E. O. McCusker, G. T. Whiteker, B. Canturk, N. Choy, L. C. Creemer, C. V. D. Amicis, N. M. Hewlett, P. L. Johnson and J. A. Knobelsdorf, *Org. Process Res. Dev.*, 2016, **20**, 661-667.
11. G. R. Fulmer, A. J. Miller, N. H. Sherden, H. E. Gottlieb, A. Nudelman, B. M. Stoltz, J. E. Bercaw and K. I. Goldberg, *Organometallics*, 2010, **29**, 2176-2179.
12. B. Sepehrnia, J. Ruble and G. Jeffrey, *Acta Crystallogr., Sect. C: Cryst. Struct. Commun.* , 1987, **43**, 249-251.
13. B. H. Bracher and R. Small, *Acta Crystallogr., Sect. C: Cryst. Struct. Commun.*, 1967, **23**, 410-418.
14. R. J. Staples and J. A. Gingold, *Z. Kristallogr. - New Cryst. Struct.*, 2009, **224**, 121-123.
15. M. Calleri, G. Chiari, A. C. Villa, A. G. Manfredotti, C. Guastini and D. Viterbo, *Acta Crystallogr., Sect. B:: Structural Crystallography and Crystal Chemistry*, 1977, **33**, 479-485.
16. M. Bursavich and F. Fronczek, *Acta Crystallogr., Sect. C: Cryst. Struct. Commun.*, 1997, **53**, IUC9700022-IUC9700022.
17. R. Laidlaw, Y. Miura, C. Panetta and R. Metzger, *Acta Crystallographica Section C: Crystal Structure Communications*, 1988, **44**, 2009-2013.
18. M. Goodman, P. Ganis, G. Avitabile and S. Migdal, *Journal of the American Chemical Society*, 1971, **93**, 3328-3331 %@ 0002-7863.
19. M. Nethaji and V. Pattabhi, 1985.
20. M. K. Singh, M. Gangwar, D. Kumar, R. Tilak, G. Nath and A. Agarwal, *Medicinal Chemistry Research*, 2014, **23**, 4962-4976 %@ 1054-2523.
21. X. Zhang, Z. Liu, Y. Gao, F. Li, Y. Tian, C. Li, X. Jia and J. Li, *Advanced Synthesis & Catalysis*, 2018, **360**, 272-277 %@ 1615-4150.
22. M. J. Barrow, S. Cradock, E. Ebsworth and D. W. Rankin, *J. Chem. Soc., Dalton Trans.*, 1981, 1988-1993.
23. A. D. Boese, M. Kirchner, G. A. Echeverria and R. Boese, *ChemPhysChem*, 2013, **14**, 799-804.
24. H. Mouhib, D. Jelisavac, W. Stahl, R. Wang, I. Kalf and U. Englert, *ChemPhysChem*, 2011, **12**, 761-764.
25. P. Baillargeon and Y. L. Dory, *Cryst. Growth Des.*, 2009, **9**, 3638-3645.
26. A. A. Yakovenko, J. H. Gallegos, M. Y. Antipin, A. Masunov and T. V. Timofeeva, *Crystal growth & design*, 2011, **11**, 3964-3978 %@ 1528-7483.
27. Y. Diskin-Posner, S. Dahal and I. Goldberg, *Chemical Communications*, 2000, 585-586.

28. Y. Diskin-Posner, G. K. Patra and I. Goldberg, *European Journal of Inorganic Chemistry*, 2001, **2001**, 2515-2523 %@ 1434-1948.
29. K. Endo, T. Ezuhara, M. Koyanagi, H. Masuda and Y. Aoyama, *Journal of the American Chemical Society*, 1997, **119**, 499-505 %@ 0002-7863.
30. P. Zou, M. H. Xie, H. Wu, Y. L. Liu and Z. P. Chen, *Acta Crystallographica Section E: Structure Reports Online*, 2011, **67**, o2806-o2806 %@ 1600-5368.
